# Glucagon‐Like Peptide‐1 Receptor Agonists Improve MASH and Liver Fibrosis: A Meta‐Analysis of Randomised Controlled Trials

**DOI:** 10.1111/liv.70256

**Published:** 2025-07-30

**Authors:** Alessandro Mantovani, Riccardo Morandin, Veronica Fiorio, Maria Giovanna Lando, Norbert Stefan, Herbert Tilg, Christopher D. Byrne, Giovanni Targher

**Affiliations:** ^1^ Metabolic Diseases Research Unit IRCCS Sacro Cuore – Don Calabria Hospital Negrar di Valpolicella Italy; ^2^ Department of Medicine University of Verona Verona Italy; ^3^ Section of Endocrinology, Diabetes and Metabolism, Department of Medicine University and Azienda Ospedaliera Universitaria Integrata of Verona Verona Italy; ^4^ Department of Internal Medicine IV, Division of Endocrinology, Diabetology and Nephrology University of Tübingen Tübingen Germany; ^5^ Institute of Diabetes Research and Metabolic Diseases of the Helmholtz Center Munich Tübingen Germany; ^6^ Department of Internal Medicine I, Gastroenterology, Hepatology, Endocrinology and Metabolism Medical University Innsbruck Innsbruck Austria; ^7^ National Institute for Health and Care Research, Southampton Biomedical Research Centre, University Hospital Southampton and University of Southampton Southampton UK

**Keywords:** GLP‐1 receptor agonists, incretin‐based therapy, metabolic dysfunction‐associated steatohepatitis, metabolic dysfunction‐associated steatotic liver disease

## Abstract

**Background/Aims:**

There is uncertainty regarding the hepatic efficacy of glucagon‐like peptide‐1 receptor agonists (GLP‐1RAs) in metabolic dysfunction‐associated steatotic liver disease (MASLD) or steatohepatitis (MASH). We performed a meta‐analysis of randomised controlled trials (RCTs) to examine the efficacy of GLP‐1RAs in treating MASLD or MASH.

**Methods:**

We systematically searched three electronic databases from inception until April 2025 to identify RCTs examining the efficacy of GLP‐1RAs for the treatment of MASLD or MASH. The outcome measures included MASH resolution without worsening of fibrosis or improvement in at least one stage of fibrosis without worsening of MASH, along with reductions in liver fat content measured using magnetic resonance‐based techniques. Meta‐analysis was conducted using random‐effects models.

**Results:**

We identified 13 phase 2 or phase 3 RCTs (1811 participants). These trials diagnosed MASLD or MASH through liver biopsy (*n* = 4) or magnetic resonance‐based techniques (*n* = 9). Regardless of diabetes status, among individuals with MASH and moderate‐to‐advanced fibrosis, GLP‐1RAs (especially semaglutide 2.4 mg/week) for up to 72 weeks were superior to placebo in achieving MASH resolution (*n* = 3 RCTs; pooled random‐effects odds ratio 3.48, 95% CI 2.69–4.51; *I* ^2^ = 0%), and in improving liver fibrosis (pooled odds ratio 1.79, 95% CI 1.37–2.35; *I* ^2^ = 0%). Among individuals with MASH‐related compensated cirrhosis (*n* = 1 RCT available only), semaglutide did not lead to MASH resolution or improved fibrosis compared to placebo. Furthermore, GLP‐1RAs reduced magnetic resonance‐measured liver fat content (*n* = 9; pooled mean difference: −4.50%, 95% CI −6.60 to −2.40%; *I* ^2^ = 95.9%).

**Conclusions:**

GLP‐1RAs are a promising treatment option for MASLD or MASH. Further research is needed to evaluate the long‐term effects of GLP‐1RAs on liver‐related clinical events.

## Summary

- In this comprehensive meta‐analysis, we examined published data from randomised controlled trials evaluating the efficacy of GLP‐1 receptor agonists for the treatment of MASLD or MASH in individuals with or without type 2 diabetes.
- GLP‐1 receptor agonists are a promising therapeutic option for MASLD or MASH. Further studies are required to examine the long‐term effects of these drugs on the risk of developing liver‐related clinical events.

## 1 Introduction

Metabolic dysfunction‐associated steatotic liver disease (MASLD), formerly known as non‐alcoholic fatty liver disease, has become the leading cause of chronic liver diseases worldwide [1, 2]. The global prevalence of MASLD is estimated to be approximately 30%–40% among adults in the general population, ~60%–70% in individuals with type 2 diabetes mellitus (T2DM), and ~70%–80% in persons with obesity [1, 2].

MASLD is a multisystem disease that creates a challenging therapeutic landscape in which pharmacotherapy must address both systemic metabolic dysfunction and liver disease to reduce the risk of developing serious liver‐related complications (such as cirrhosis, hepatic decompensation events, and hepatocellular carcinoma) and extrahepatic cardiometabolic outcomes (cardiovascular disease, chronic kidney disease, and new‐onset T2DM) [3, 4, 5].

In March 2024, resmetirom (a liver‐directed thyroid hormone receptor beta‐selective agonist) became the first drug to receive conditional approval from the US Food and Drug Administration for treating adults with non‐cirrhotic metabolic dysfunction‐associated steatohepatitis (MASH) and moderate‐to‐advanced liver fibrosis [6, 7]. However, resmetirom has a neutral impact on body weight and insulin resistance, and its long‐term effect on the risk of major adverse cardiovascular events is currently unknown [8]. Furthermore, the relatively limited efficacy of resmetirom in improving liver fibrosis and its restricted global availability since approval highlight the need for other therapeutic options for MASLD/MASH.

Since the pathogenesis of MASLD/MASH is closely linked to metabolic dysfunction and insulin resistance, and cardiovascular disease is the leading cause of death in people with MASLD/MASH [5], it is believed that agents improving the cardiometabolic risk profile could also improve MASLD/MASH‐related outcomes [4].

Large cardiovascular and renal outcome trials have demonstrated the effectiveness of glucagon‐like peptide‐1 receptor agonists (GLP‐1RAs) in reducing the risk of overall mortality and adverse cardiovascular and renal outcomes [9, 10, 11, 12]. Additionally, some meta‐analyses of Phase 2 placebo‐controlled or active‐comparator‐controlled randomised controlled trials (RCTs) have shown that GLP‐1RA treatment can reduce liver fat content (as measured by magnetic resonance‐based techniques) and is associated with histological resolution of MASH, although it does not show improvement in liver fibrosis in MASLD/MASH [13, 14]. While the benefit of GLP‐1RAs on liver fibrosis—that is, the strongest histological predictor of mortality and liver‐related and extrahepatic complications in MASLD—remains uncertain, these findings support the use of GLP‐1RAs in people living with MASLD who have cardiometabolic risk factors.

Understanding the hepatic effectiveness of GLP‐1RAs on histological endpoints is critically important, given the increasing use of these antihyperglycaemic agents in populations at high risk for liver‐related and cardiometabolic outcomes. Results from long‐term Phase 3 placebo‐controlled RCTs assessing the efficacy of GLP‐1RAs are ongoing. Recently, Sanyal et al. reported results from part 1 of the ESSENCE trial, a Phase 3 placebo‐controlled randomised trial involving 800 adults with biopsy‐proven MASH and liver fibrosis. This RCT examined the 72‐week effect of once‐weekly subcutaneous semaglutide 2.4 mg versus placebo on MASH resolution without worsening of fibrosis and improvement in fibrosis without worsening of MASH [15].

Therefore, in this updated and comprehensive systematic review and meta‐analysis, we examined the published data from Phase 2 and Phase 3 RCTs that evaluate the efficacy of GLP‐1RAs for the treatment of MASLD or MASH in individuals with or without pre‐existing T2DM.

## 2 Materials and Methods

### 2.1 Protocol Registration

The protocol of the meta‐analysis was registered in advance on Open Science Framework registries (https://doi.org/10.17605/OSF.IO/HGVS4).

### 2.2 Data Sources and Searches

We conducted this systematic review and meta‐analysis following the Preferred Reporting Items for Systematic Reviews and Meta‐Analyses (PRISMA) guidelines. We systematically searched the PubMed, Web of Science, and ClinicalTrials.gov databases from their inception to April 30, 2025, to identify RCTs evaluating the efficacy of GLP‐1RAs in the treatment of MASLD or MASH. The primary search terms (based on both MeSH and free‐text terms) included “non‐alcoholic fatty liver disease” (OR “NAFLD” OR “non‐alcoholic steatohepatitis” OR “NASH”) OR “metabolic dysfunction‐associated steatotic liver disease” (OR “MASLD” OR “metabolic dysfunction‐associated steatohepatitis” OR “MASH”) OR “metabolic dysfunction‐associated fatty liver disease” (OR “MAFLD”) AND “glucagon‐like peptide‐1 receptor agonists” OR “GLP‐1 receptor agonists” OR “exenatide” OR “liraglutide” OR “lixisenatide” OR “albiglutide” OR “dulaglutide” OR “semaglutide”. Searches were limited to human studies without language restrictions.

### 2.3 Inclusion and Exclusion Criteria

Studies were included if they were Phase 2 or Phase 3 placebo‐controlled or active‐comparator‐controlled RCTs involving adult (≥ 18 years old) individuals with MASLD or MASH (regardless of their T2DM status) that examined the efficacy of GLP‐1RAs on MASLD or MASH. The diagnosis of MASLD/MASH was based on liver biopsy or magnetic resonance‐based techniques, such as magnetic resonance imaging‐proton density fat fraction (MRI‐PDFF) or magnetic resonance spectroscopy (MRS). Among the active‐comparator‐controlled RCTs, we included only trials with an active antihyperglycaemic drug as a control arm for comparison (specifically those involving participants with T2DM). We excluded active‐comparator‐controlled RCTs that had a control arm consisting of non‐antihyperglycaemic drugs tested for their potential hepatoprotective effects (e.g., fibroblast growth factor‐21 [FGF‐21] analogues, and farnesoid X receptor [FXR] agonists—see Table S1). We also excluded RCTs using dual or triple incretin receptor agonists, such as tirzepatide, survodutide, cotadutide, efinopegdutide, pemvidutide or retatrutide. Additionally, we excluded RCTs in which the diagnosis of MASLD/MASH was based on methods other than liver biopsy or magnetic resonance‐based techniques. Finally, we excluded case reports, retrospective observational studies, and non‐randomised interventional studies.

### 2.4 Data Extraction and Quality Assessment

Two investigators (AM and GT) independently reviewed the titles and abstracts of all RCTs identified using the above‐mentioned inclusion criteria. Each RCT that met the initial inclusion criteria underwent a full‐text review by both investigators independently. Any disagreements about the inclusion of studies were resolved by a third independent investigator (RM).

For each eligible RCT, we extracted data on the publication year, study country, sample size, participant characteristics (i.e., age, sex, ethnicity, Body Mass Index [BMI], pre‐existing T2DM, and serum liver enzyme levels), follow‐up duration, type of intervention, dosages of GLP‐1RAs or active drug comparators, and methods used for diagnosing MASLD/MASH.

The risk of bias for each eligible RCT was assessed using the Cochrane Collaboration tool by two independent authors (AM and GT). Any disagreements in scoring were reviewed, and a consensus was reached after discussion. The Cochrane Collaboration's tool assesses seven potential sources of bias: random sequence generation (selection bias), allocation concealment (selection bias), blinding of participants and personnel (performance bias), blinding of outcome assessment (detection bias), incomplete outcome data (attrition bias), selective reporting (reporting bias), and other bias. For each of these domains, we categorised each eligible RCT into three levels: low, unclear or high risk of bias [16].

### 2.5 Data Synthesis and Analysis

The primary outcome measures were changes in the percentage of resolution of MASH without worsening liver fibrosis or an improvement in at least one stage of liver fibrosis without worsening of MASH. As secondary outcome measures, we collected data on changes in the absolute percentage of liver fat content using magnetic resonance techniques, as well as changes in mean post‐treatment values of serum liver enzyme levels, haemoglobin A1c and body weight.

For each RCT, the effect sizes of the primary outcome measures between participants randomly assigned to GLP‐1RA treatment and those assigned to placebo or reference therapy are presented as odds ratios (OR) and 95% confidence intervals (CI) for histological resolution of MASH or ≥ 1‐stage fibrosis improvement using both fixed‐ and random‐effect models. For changes in liver fat content (assessed by MRI‐based techniques), serum liver enzyme levels, haemoglobin A1c and body weight, the effect sizes are presented as mean differences (MD) and 95% CIs [17].

To evaluate the robustness of the observed associations, we conducted subgroup analyses based on baseline MASH‐related cirrhosis status (for RCTs using liver biopsy), study country, and type of comparator drug in the control arm (for RCTs using magnetic resonance imaging). We also performed univariable meta‐regression analyses to examine the potential effects of sex, age, body mass index, proportion of pre‐existing T2DM, and percentage changes in body weight during the trial on the observed GLP‐1RA‐induced reduction in the absolute percentage of liver fat content, as measured by MRI‐PDFF or MRS. We also tested for the possible excessive influence of individual studies using a meta‐analysis influence (leave‐one‐out) test, which eliminated each included RCT one at a time.

A visual inspection of the forest plots was conducted to evaluate the presence of statistical heterogeneity [17]. Statistical heterogeneity between studies was assessed using the chi‐square test and the *I* ^2^‐statistic, which estimates the percentage of variability between studies attributable to heterogeneity rather than chance alone [18]. The proportion of heterogeneity explained by between‐study variability was evaluated using the *I* ^2^‐statistic and considered low if the *I* ^2^‐index was 0% to 40%, or substantial if the *I* ^2^‐index was greater than 50% [18]. Publication bias was assessed through visual inspection of funnel plots and the Egger regression test [17].

All statistical tests were two‐sided, and a *p*‐value < 0.05 was considered significant. We used R software (version 4.2.2/2022) for all statistical analyses with the following packages: *meta* (version 8.0–1) and *metafor* (version 4.6–0).

### 2.6 Funding Source

There was no funding source for this meta‐analysis.

## 3 Results

### 3.1 Search Results and Study Characteristics

The PRISMA flow diagram summarises the search and selection processes of the meta‐analysis (Figure S1). After removing duplicates and screening titles and abstracts, we identified 16 studies from the PubMed, Scopus, and ClinicalTrials.gov databases for eligibility assessment. Subsequently, we excluded three studies due to unsatisfactory inclusion criteria, as specified in Table S1. In total, 13 Phase 2 or Phase 3 RCTs met the eligibility criteria and were included in the final analysis [15, 19, 20, 21, 22, 23, 24, 25, 26, 27, 28, 29, 30].

The main characteristics of these 13 Phase 2 or Phase 3 RCTs (including eleven placebo‐controlled and two active‐comparator‐controlled trials) are detailed in Table 1. In total, 1811 middle‐aged overweight or obese individuals with MASLD or MASH were included in the study (> 75% White individuals; 43% men; 64% had known T2DM; mean age 52 years; mean BMI 33 kg/m^2^; mean serum aspartate aminotransferase (AST) 38 IU/L; mean serum alanine aminotransferase (ALT) 49 IU/L). These participants were treated for a median duration of 26 weeks (interquartile range: 24–72 weeks), with a median of 72 weeks for RCTs with liver biopsy data and a median of 26 weeks for RCTs using MRI‐based data, respectively.

**TABLE 1:** Principal randomised clinical trials of GLP‐1RAs for the treatment of MASLD or MASH (eleven placebo‐controlled RCTs and two active‐comparator‐controlled RCTs) ordered by diagnostic methodology used for MASLD/MASH and publication year.

Author, Year, Country (Reference)	RCT's characteristics	Interventions (*n*), RCT's length	Efficacy and/or effectiveness outcomes A vs. B (or vs. C or D)	Major adverse effects
**Magnetic resonance‐based clinical trials**				
Dutour et al. 2016; France [19]	Patients with T2DM, 95% of whom had MASLD on MRS (phase 2a placebo‐controlled RCT) White ethnicity: 100%; mean age: 52 years; male sex: 48%; BMI 36 kg/m^2^; HbA1c 7.5%; ALT 29 IU/L; AST 22 IU/L	A: Exenatide 5–10 mcg bid (*n* = 22) B: Placebo (*n* = 22) Length: 26 weeks	Exenatide and reference treatment led to a similar improvement in HbA1c (−0.7% ± 0.3% vs. −0.7% ± 0.4%; *p* = 0.29) Significant weight loss was observed in the exenatide group (−5.5 ± 1.2 kg vs. −0.2 ± 0.8 kg; *p* = 0.001 for difference between groups) Exenatide induced a significant reduction in liver fat content, compared with the reference treatment (liver fat content: −23.8% ± 9.5% vs. +12.5% ± 9.6%, *p* = 0.007)	Not reported
Frossing et al. 2018; Denmark [20]	Women without diabetes with polycystic ovary syndrome and MASLD on MRS (phase 2a placebo‐controlled RCT) White ethnicity: 100%; mean age: 47 years; female sex: 100%; BMI 33 kg/m^2^	A. Liraglutide 1.8 mg/day (*n* = 48) B. Placebo (*n* = 24) Length: 26 weeks	Liraglutide reduced body weight by 5.2 kg (−5.6% from baseline), liver fat content (on MR spectroscopy) by 44%, and the prevalence of MASLD by about two‐thirds (all *p* < 0.01) Liraglutide caused significantly larger reductions in fasting glucose (liraglutide vs. placebo, mean between‐group difference [95% CI], −0.24 [−0.44 to −0.04] mmol/L; mean HbA1c [95% CI], −1.38 [−2.48 to −0.28] mmol/mol)	Nausea (79% liraglutide vs. 13% placebo) and constipation (26% liraglutide vs. 0% placebo) were the most prevalent adverse events
Yan et al. 2019; China [21]	Patients with T2DM and MASLD on MRI‐PDFF (phase 2a active drug‐controlled RCT) Asian ethnicity: 100%; mean age: 44 years; male sex: 69%; BMI 29.8 kg/m^2^; HbA1c 7.7%; ALT 43 IU/L; AST 33 IU/L	A. Liraglutide 1.8 mg/day (*n* = 24) B. Insulin glargine 0.2 IU/kg/day (*n* = 24) C. Sitagliptin 100 mg/day (*n* = 27) Length: 26 weeks	In the liraglutide and sitagliptin groups, liver fat content significantly decreased from baseline to week 26 (liraglutide, 15.4% ± 5.6% to 12.5% ± 6.4%, *p* < 0.001; and sitagliptin, 15.5% ± 5.6% to 11.7% ± 5.0%, *p* = 0.001) but not in the insulin glargine group HbA1c decreased in all treatment groups (liraglutide, 7.8% ± 1.4% to 6.8% ± 1.7%, *p* < 0.001; sitagliptin, 7.6% ± 0.9% to 6.6% ± 1.1%, *p* = 0.016; and insulin glargine, 7.7% ± 0.9% to 6.9% ± 1.1%, *p* = 0.013) Body weight significantly decreased in the liraglutide and sitagliptin groups (but not in the insulin glargine group)	Not reported
Khoo et al. 2019; Singapore [22]	Patients without diabetes with obesity and MASLD on MRI‐PDFF (phase 2a placebo‐controlled RCT) Asian ethnicity: 100%; mean age: 41 years; male sex: 90%; BMI 33 kg/m^2^; ALT 88 IU/L; AST 48 IU/L	A. Liraglutide 3.0 mg/day (*n* = 15) B. Lifestyle intervention (diet+exercise) (*n* = 15) Length: 26 weeks	The two treatment groups had significant (*p* < 0.01) and similar reductions in liver fat content (−8.1 ± 13.2 vs. −7.0% ± 7.1%), serum ALT (−39 ± 35 vs. −26 ± 33 U/L) and body weight at 26 weeks	Nausea (80% vs. 0%), abdominal discomfort (100% vs. 6.7%) and diarrhoea (33% vs. 0%) were more frequent in the liraglutide group than in placebo
Liu et al. 2020; China [23]	Patients with T2DM and MASLD on MRI‐PDFF (phase 2a active drug‐controlled RCT) Asian ethnicity: 100%; mean age: 48 years; male sex: 50%; BMI 28 kg/m^2^; HbA1c 8.3%; ALT 38 IU/L; AST 28 IU/L	A. Exenatide 5–10 mcg bid (*n* = 38) B. Insulin glargine 0.1–0.3 IU/kg/day (*n* = 38) Length: 24 weeks	With exenatide treatment, liver fat content was significantly reduced (Δliver fat: −17.6% ± 13%). Exenatide treatment also resulted in greater reductions in visceral adipose tissue decreased in the exenatide group (ΔVAT: −43.6 ± 68 cm^2^) compared to the control group, and in serum ALT, AST, GGT levels, BMI and waist circumference than in the control group	The proportion of adverse events was comparable between the two groups (13.2% for exenatide vs. 15.8% for insulin glargine)
Bizino et al. 2020; Netherlands [24]	Patients with T2DM and MASLD on MRS (phase 2a placebo‐controlled RCT) White ethnicity: 100%; mean age: 60 years; male sex: 59%; BMI 32 kg/m^2^; HbA1c 8.3%; ALT 14 IU/L; AST 33 IU/L	A. Liraglutide 1.8 mg/day (*n* = 23) B. Placebo (*n* = 26) Length: 26 weeks	Change in liver fat content was not different between the groups (liraglutide 18.1% ± 11.2% to 12.0% ± 7.7%; placebo 18.4% ± 9.4% to 14.7% ± 10.0%; estimated treatment effect −2.1 [95% CI −5.3, 1.0]%) Liraglutide vs. placebo significantly reduced body weight (liraglutide 98.4 ± 13.8 kg to 94.3 ± 14.9 kg; placebo 94.5 ± 13.1 kg to 93.9 ± 3.2 kg; estimated treatment effect −4.5 [95% CI −6.4, −2.6] kg) Serum liver enzymes and HbA1c levels declined in both groups without a significant treatment effect of liraglutide vs. placebo (liraglutide HbA1c 8.4% ± 1.1% to 7.3% ± 1.2%; placebo HbA1c 8.2% ± 1.0% to 7.5% ± 0.7%)	There were no serious drug‐related adverse events
Kuchay et al. 2020; India [25]	Patients with T2DM and MASLD on MRI‐PDFF (open‐label trial add‐on to usual care: the D‐LIFT trial) Asian ethnicity: 100%; mean age: 47 years; male sex: 70%; BMI 29.7 kg/m^2^; HbA1c 8.4%; ALT 69 IU/L; AST 47 IU/L	A. Dulaglutide 1.5 mg/week (*n* = 32) B. Placebo (*n* = 32) Length: 24 weeks	Dulaglutide resulted in a control‐corrected absolute change in liver fat content of −3.5% (95% CI −6.6, −0.4; *p* = 0.025) and a relative change of −26.4% (−44.2, −8.6; *p* = 0.004) Dulaglutide resulted in a significantly larger reduction in serum GGT levels (mean between‐group difference −13.1 U/L [95% CI −24.4, −1.8]; *p* = 0.025) and in non‐significant changes in serum AST and ALT levels Absolute changes in liver stiffness on Fibroscan (liver stiffness: −1.31 kPa [−2.99, 0.37]; *p* = 0.12) were not significant when comparing the two groups	There were no serious drug‐related adverse events
Guo et al. 2020; China [26]	Patients with T2DM and MASLD on MRS (open‐label trial add‐on to usual care) Asian ethnicity: 100%; mean age: 52 years; male sex: 60%; BMI 28.6 kg/m^2^; HbA1c 7.4%; ALT 32 IU/L; AST 28 IU/L	A. Liraglutide 1.8 mg/day (*n* = 31) B. Insulin glargine 0.1–0.3 IU/kg/day (*n* = 30) C. Placebo (*n* = 30) Length: 26 weeks	Liraglutide resulted in a significant decrease in liver fat content compared to placebo (liraglutide: 26.4% ± 3.2% to 20.6% ± 3.9%; placebo: 25.8% ± 4.5% to 25.7% ± 3.6%, *p* < 0.05) There was also a decrease in liver fat content in the insulin glargine group, but it was not statistically significant from baseline to week 26 Dulaglutide resulted in significant reductions in body weight (including visceral adipose tissue) and serum ALT and AST levels	Nausea and vomiting (0% placebo vs. 6.7% insulin glargine vs. 25.8% liraglutide) were the most prevalent adverse events
Flint et al. 2021; Germany [27]	Patients with/without T2DM and with MASLD on MRI‐PDFF and MRI‐elastography (phase 2a placebo‐controlled RCT) White ethnicity: 100%; mean age: 60 years; male sex: 70%; BMI 35 kg/m^2^; HbA1c 7.3% (for diabetics); ALT 37 IU/L; AST 30 IU/L	A. Semaglutide 0.4 mg/day (*n* = 34) B. Placebo (*n* = 33) Length: 72 weeks	At week 72, reductions in liver fat content were significantly greater with semaglutide (estimated treatment ratio: 0.50 [0.39, 0.66], *p* < 0.0001), and more subjects achieved ≥ 30% reduction in liver fat content with semaglutide at weeks 24, 48 and 72 (all *p* < 0.001) Changes from baseline in liver stiffness were not significantly different between semaglutide and placebo at week 72 Semaglutide decreased serum liver enzymes, HbA1c and body weight	Gastrointestinal AEs were reported by more patients in the semaglutide than the placebo group, with diarrhoea (30.3% vs. 24.2%) and nausea (30.3% vs. 9.1%) the most frequent
**Liver biopsy‐based clinical trials**				
Armstrong et al. 2016; United Kingdom [28]	Patients with MASH and fibrosis stages F2‐4 on liver biopsy (phase 2b placebo‐controlled RCT: the LEAN trial) White ethnicity: 88%; mean age: 51 years; male sex: 60%; BMI 36 kg/m^2^; ALT 71 IU/L; AST 51 IU/L; fibrosis F3‐F4 (on histology) 52%; pre‐existing T2DM: 33%	A. Liraglutide 1.8 mg/day (*n* = 26) B. Placebo (*n* = 26) Length: 48 weeks	Histologic resolution of MASH in the liraglutide vs. placebo groups: 39% vs. 9%, *p* = 0.019 Change in histologic NAS score in the liraglutide vs. placebo groups: −1.3 vs. −0.8, *p* = 0.24 Change in fibrosis stage in the liraglutide vs. placebo groups: −0.2 vs. 0.2, *p* = 0.11 Fibrosis improvement in the liraglutide vs. placebo groups: 26% vs. 14%, *p* = 0.46 Fibrosis worsening in the liraglutide vs. placebo groups: 9% vs. 36%, *p* = 0.04 Change in serum ALT in the liraglutide vs. placebo groups: −26.6 vs. −10.2 UI/L, *p* = 0.16 Change in serum AST in the liraglutide vs. placebo groups: −27 vs. + 9 IU/L; *p* = 0.02	Moderate gastrointestinal disorders in the liraglutide vs. placebo: 81% vs. 65%. In particular, nausea (46% vs. 38%), diarrhoea (38% vs. 19%) and vomiting (19% vs. 12%) were the most frequent adverse events in the liraglutide group compared to placebo
Newsome et al. 2021; International cohort of individuals from 16 countries [29]	Patients with MASH and fibrosis (stages F1‐3) on liver biopsy (phase 2b placebo‐controlled RCT) White ethnicity: 77%; mean age: 55 years; male sex: 41%; BMI 35.7 kg/m^2^; pre‐existing T2DM: 62% (HbA1c 7.3%); ALT 54 IU/L; AST 43 IU/L	A. Semaglutide 0.1 mg/day (*n* = 80) B. Semaglutide 0.2 mg/day (*n* = 78) C. Semaglutide 0.4 mg/day (*n* = 82) D. Placebo (*n* = 80) Length: 72 weeks	Percentage of patients in whom MASH resolution was achieved with no worsening of fibrosis was 40% in the 0.1‐mg group, 36% in the 0.2‐mg group, 59% in the 0.4‐mg group, and 17% in the placebo group (*p* < 0.001 for semaglutide 0.4 mg vs. placebo) Improvement in fibrosis stage occurred in 43% of the patients in the 0.4‐mg group and 33% of the patients in the placebo group (*p* = 0.48) Treatment with semaglutide resulted in dose‐dependent reductions in serum ALT and AST levels Mean percent weight loss was 13% in the 0.4‐mg group and 1% in the placebo group (*p* < 0.001)	The percentages of patients with nausea, constipation, decreased appetite, vomiting, and abdominal pain were higher in the semaglutide 0.4‐mg group than in the placebo group (nausea, 42% vs. 11%; constipation, 22% vs. 12%; decreased appetite, 22% vs. 5%; vomiting, 15% vs. 2%; and abdominal pain, 7% vs. 4%)
Loomba et al. 2023; International cohort of individuals from 38 centres in Europe and the USA [30]	Patients with MASH‐related compensated cirrhosis (stage F4) on liver biopsy (phase 2b placebo‐controlled RCT) White ethnicity: 87%; mean age: 59.5 years; male sex: 31%; BMI 34.9 kg/m^2^; pre‐existing T2DM: 75% (HbA1c 7.2%); ALT 42 IU/L; AST 44 IU/L	A. Semaglutide 2.4 mg/week (*n* = 47) B. Placebo (*n* = 24) Length: 48 weeks	At week 48, there was no significant difference between the two groups in the proportion of patients with an improvement in liver fibrosis of one stage or more without worsening of MASH (11% in the semaglutide group vs. 29% in the placebo group); odds ratio 0.28 (95% CI 0.06–1.24; *p* = 0.087) There was no significant difference between groups in the proportion of patients who achieved MASH resolution (*p* = 0_·_29) Despite the lack of histological changes with semaglutide, improvements were seen in liver fat content by MRI‐PDFF as well as non‐invasive markers of liver fat and liver injury associated with fibrosis progression	Similar proportions of patients in each group reported adverse events. The most common adverse events were nausea (45% vs. 17%), diarrhoea (19% vs. 8%), and vomiting (17% vs. none)
Sanyal et al. 2025; International cohort of individuals from 37 countries [15]	Patients with MASH and fibrosis (stages F2 or F3) on liver biopsy (phase 3 placebo‐controlled RCT: the ESSENCE trial) White ethnicity: 67%; mean age: 56 years; male sex: 43%; BMI 34.6 kg/m^2^; pre‐existing T2DM: 55% (HbA1c 7.2%); ALT 68 IU/L; AST 53 IU/L	A. Semaglutide 2.4 mg/week (*n* = 534) B. Placebo (*n* = 266) Length: 72 weeks	Percentage of patients in whom MASH resolution was achieved with no worsening of fibrosis was 62.9% in the semaglutide group and 34.3% in the placebo group (*p* < 0.001 for semaglutide vs. placebo) Improvement in at least one stage of liver fibrosis occurred in 36.8% of the patients in the semaglutide group and 22.4% of those in the placebo group (*p* < 0.001) Semaglutide resulted in significant reductions in serum ALT and AST levels and non‐invasive fibrosis tests, as well as body weight and HbA1c compared to placebo	Nausea (36% vs. 13%), diarrhoea (27% vs. 12%), constipation (22% vs. 8%), and vomiting (18.6% vs. 5.6%) were higher in the semaglutide group than in the placebo group

*Note:* In most randomised clinical trials included, MASLD/MASH were originally labelled as NAFLD or NASH.

Abbreviations: ALT, alanine aminotransferase; AST, aspartate aminotransferase; BMI, body mass index; GGT, gamma‐glutamyltransferase; MRS, magnetic resonance spectroscopy; MRI‐PDFF, magnetic resonance imaging‐proton density fat fraction; MASLD, metabolic dysfunction‐associated steatotic liver disease; MASH, metabolic dysfunction‐associated steatohepatitis; RCT, randomised controlled trial; T2DM, type 2 diabetes mellitus.

Participants who met the criteria were randomly assigned to receive subcutaneous exenatide (*n* = 2 RCTs), liraglutide (*n* = 6 RCTs), dulaglutide (*n* = 1 RCT), semaglutide (*n* = 4 RCTs), or placebo/reference therapy specifically to treat MASLD or MASH. The diagnosis of MASLD was based on liver biopsy in four RCTs that included individuals with MASH and liver fibrosis (stages F2 to F4), while the remaining nine RCTs utilised MRI‐based techniques (MRI‐PDFF or MRS). Most of these RCTs specifically included individuals with T2DM (*n* = 6 RCTs), while five RCTs included participants with and without T2DM, and two RCTs were conducted in individuals who did not have T2DM, particularly those with obesity or women with polycystic ovary syndrome. Two were active‐comparator‐controlled RCTs involving patients with T2DM that compared GLP‐1 RA use versus sitagliptin and/or insulin glargine. Three RCTs included multinational cohorts (recruited in Europe, the United States, and other countries), five RCTs were conducted in Asia (China, Singapore, and India), and five were conducted in Europe (the United Kingdom, France, Denmark, Germany, and the Netherlands). Among the eligible RCTs with available data on adverse effects, GLP‐1RAs were generally well‐tolerated and had a similar adverse event profile to either placebo or reference therapy, except for a higher frequency of gastro‐intestinal symptoms, such as nausea, vomiting, constipation, diarrhoea, or abdominal discomfort. However, these gastro‐intestinal symptoms were mainly transient and mild‐to‐moderate in severity across the included RCTs. According to the Cochrane Collaboration's tool, eligible RCTs were assessed to have a low risk of bias (*n* = 5) or a moderate risk of bias (*n* = 8); no studies were considered to have a high risk of bias (as illustrated in Table S2).

### 3.2 Effect of GLP‐1RAs on Liver Histological Endpoints

**FIGURE 1:**
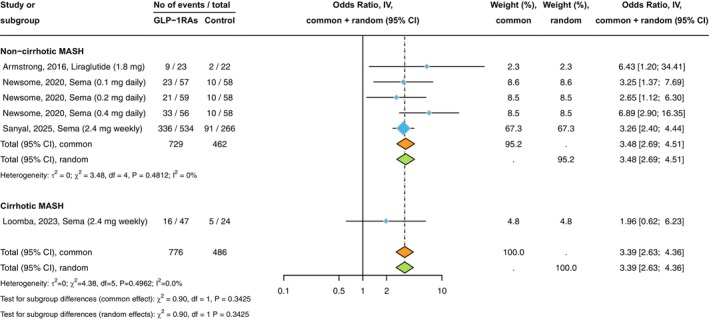
Forest plot and pooled estimates of the effect of GLP‐1RAs on the histologic resolution of MASH without worsening of liver fibrosis, compared to placebo, in RCTs stratified by the presence of MASH‐related cirrhosis at baseline (*n* = 4 placebo‐controlled RCTs).

Figure 1 shows the forest plot and pooled estimates of the effect of GLP‐1RAs on the histologic resolution of MASH without worsening fibrosis. These findings are based on four placebo‐controlled RCTs involving 1262 participants randomly assigned to placebo or treatment with liraglutide 1.8 mg/day, semaglutide 2.4 mg/week, or semaglutide at doses of 0.1, 0.2, and 0.4 mg/day administered subcutaneously. When analysing all these RCTs together, GLP‐1 RA treatment significantly increased the rate of MASH resolution compared to placebo (*n* = 4 RCTs; pooled random‐effects odds ratio 3.39, 95% CI 2.63–4.36, *p* < 0.001; *I* ^2^ = 0%). Notably, after stratifying RCTs for MASH‐related cirrhosis status at baseline, we found that in patients with MASH and liver fibrosis (stages F2 or F3), GLP‐1RA treatment for up to 72 weeks was superior to placebo in achieving MASH resolution without worsening of fibrosis (*n* = 3 RCTs; pooled odds ratio 3.48, 95% CI 2.69–4.51, *p* < 0.001; *I* ^2^ = 0%). This beneficial effect of GLP‐1RAs on MASH resolution remained significant even after excluding the Phase 3 RCT by Sanyal et al. (pooled odds ratio 4.07, 95% CI 2.44–6.79, *p* < 0.001; *I* ^2^ = 0%). Conversely, in patients with MASH‐related compensated cirrhosis (*n* = 1 RCT involving 71 individuals), once‐weekly semaglutide 2.4 mg for 48 weeks did not achieve MASH resolution compared to placebo (pooled odds ratio 1.96, 95% CI 0.62–6.23).

**FIGURE 2:**
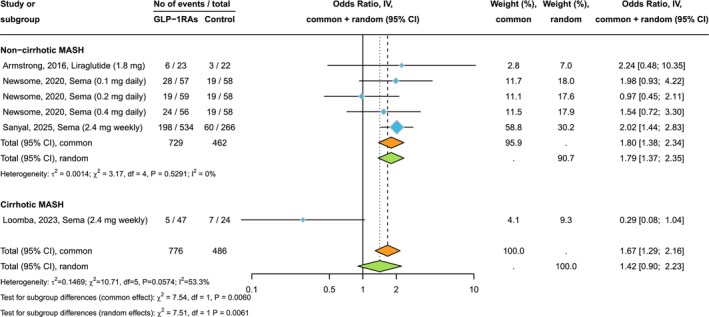
Forest plot and pooled estimates of the effect of GLP‐1RAs on improvement in ≥ 1‐stage liver fibrosis without worsening of MASH compared to placebo, in RCTs stratified by the presence of MASH‐related cirrhosis at baseline (*n* = 4 placebo‐controlled RCTs).

Figure 2 shows the forest plot and pooled estimates of the effect of GLP‐1RAs on the improvement of at least one stage of liver fibrosis without worsening of MASH. In patients with MASH and liver fibrosis, treatment with GLP‐1RAs (primarily semaglutide) for up to 72 weeks was more effective than placebo in improving liver fibrosis without worsening of MASH (pooled odds ratio 1.79, 95% CI 1.37–2.35, *p* < 0.001; *I* ^2^ = 0%). However, this beneficial effect of GLP‐1RAs on fibrosis improvement was lost after removing the Phase 3 RCT by Sanyal et al. from the analysis (pooled odds ratio 1.50, 95% CI 0.98–2.28, *p* = 0.062; *I* ^2^ = 0%). In patients with MASH‐related compensated cirrhosis, treatment with semaglutide for 48 weeks did not lead to fibrosis improvement (pooled odds ratio 0.29, 95% CI 0.08–1.04).

### 3.3 Effect of GLP‐1RAs on MRI‐Assessed Liver Fat Content

**FIGURE 3:**
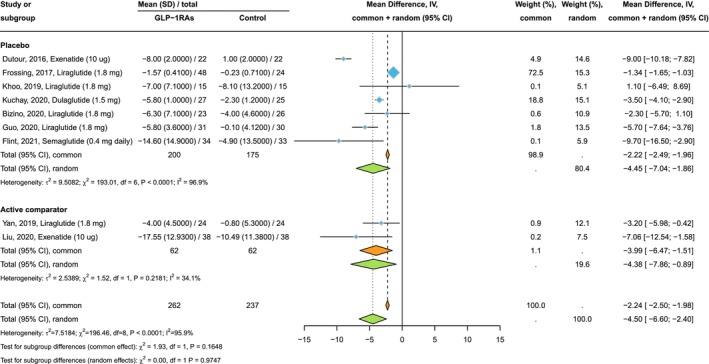
Forest plot and pooled estimates of the effect of GLP‐1RAs on the absolute percentage of liver fat content, assessed by magnetic resonance‐based techniques, in RCTs stratified by the type of comparator drug (*n* = 7 placebo‐controlled and *n* = 2 active‐comparator‐controlled trials).

Figure 3 shows the forest plot and pooled estimates of the effects of GLP‐1RAs on liver fat content, assessed via MRI‐PDFF or MRS (*n* = 9 RCTs involving 499 individuals who were randomly assigned to either placebo/reference therapy or to exenatide 10 mcg/day, liraglutide 1.8 mg/day, dulaglutide 1.5 mg/week, or semaglutide at 0.4 mg/day subcutaneously). Compared to placebo or reference therapy, treatment with GLP‐1RAs for up to 26 weeks was associated with a significant reduction in the absolute percentage of liver fat content (pooled mean difference (MD): −4.50%, 95% CI −6.60 to −2.40%; *p* < 0.001; *I* ^2^ = 95.9%). This absolute percentage reduction corresponds to a decrease in the mean relative percentage change in liver fat content of −35% for GLP‐1RAs compared to −14% for placebo, respectively. Similar results were observed when we examined the effect of GLP‐1RAs on liver fat reduction in placebo‐controlled and active‐comparator‐controlled RCTs, separately.

### 3.4 Effects of GLP‐1RAs on Serum Liver Enzymes, Haemoglobin A1c and Body Weight

Figures S2–S4 show the forest plots and pooled estimates of the effect of GLP‐1RAs on mean post‐treatment values of serum liver enzymes. Compared to placebo or reference therapy, GLP‐1RA use was associated with significant reductions in circulating levels of ALT (pooled MD: −9.99 IU/L, 95% CI −16.8 to −3.18 IU/L), AST (pooled MD: −7.03 IU/L, 95% CI −13.43 to −0.63 IU/L) and gamma‐glutamyltransferase (GGT) (pooled MD: −18.29 IU/L, 95% CI −29.26 to −7.32 IU/L).

Figures S5 and S6 show the forest plots and pooled estimates of the effect of GLP‐1RAs on mean post‐treatment values of body weight and HbA1c levels. Compared to placebo/reference therapy, treatment with GLP‐1RAs was associated with significant reductions in body weight (pooled MD: −4.48 kg, 95% CI −6.08 to −2.88 kg, *p* < 0.001) and HbA1c levels (pooled MD: −1.30%, 95% CI −1.69% to −0.91%).

### 3.5 Subgroup and Meta‐Regression Analyses

A subgroup analysis by study country revealed that the observed GLP‐1RA‐induced reduction in liver fat content (as measured with MRI‐based techniques) was consistent in RCTs conducted in both Europe and Asian countries (Figure S7). We also conducted univariable meta‐regression analyses to explore the potential influence of moderator variables on the observed reduction in liver fat content, expressed as absolute percentage and measured by MRI‐PDFF or MRS (Figures S8–S12). These meta‐regression analyses suggested that sex, age, BMI, pre‐existing T2DM, and percentage changes in body weight during the trial did not significantly affect the effect size for the GLP‐1RA‐induced reduction in liver fat content, although the influence of body weight change during the trial was marginally significant (*p* = 0.059). Finally, we also conducted a leave‐one‐out meta‐analysis to examine the influence of each RCT on the overall effect size estimate for hepatic histological endpoints and MRI‐measured liver fat content and to identify potential influential studies (Figure S13).

### 3.6 Publication Bias Testing

As shown in Figures S14 and S15, the funnel plots were quite symmetrical. The Egger's regression test did not indicate any statistically significant asymmetry in the funnel plots of the RCTs examining the effects of GLP‐1RAs on MASH resolution (*p* = 0.703) or improvement in liver fat content (*p* = 0.151), as evaluated by MRI‐based techniques. Although the number of RCTs included was fewer than 10, these results suggest that publication bias was low.

## 4 Discussion

To our knowledge, this is the largest and most comprehensive meta‐analysis of Phase 2 or Phase 3 RCTs that used GLP‐1RAs for the treatment of MASLD or MASH. The meta‐analysis incorporated 13 placebo‐controlled (*n* = 11) or active‐comparator‐controlled (*n* = 2) RCTs from different countries (including the recent Phase 3 ESSENCE trial) that aggregated data on 1811 middle‐aged individuals with overweight or obesity for whom the diagnosis of MASLD was based on magnetic resonance‐based techniques, and MASH±liver fibrosis was assessed histologically [15]. Most individuals included in these RCTs had comorbid T2DM, accounting for ~65% of participants.

The main and novel findings of this meta‐analysis indicate that among individuals with MASH and liver fibrosis (stages F2 or F3), treatment with GLP‐1RAs for up to 72 weeks was better than placebo in achieving MASH resolution without worsening of fibrosis (*n* = 3 placebo‐controlled RCTs; pooled odds ratio 3.48, 95% CI 2.69–4.51; *I* ^2^ = 0%) and also in improving at least one stage of liver fibrosis without worsening of MASH (pooled odds ratio 1.79, 95% CI 1.37–2.35; *I* ^2^ = 0%). In contrast, for individuals with MASH‐related compensated cirrhosis (only one small phase 2 RCT available), treatment with semaglutide for 48 weeks did not improve liver fibrosis or lead to MASH resolution compared with placebo. However, larger RCTs with longer follow‐up durations are needed to confirm this finding. Additionally, GLP‐1RA use for up to 26 weeks was associated with significant reductions in liver fat content, measured by MRI‐based techniques (*n* = 9 RCTs; pooled mean difference: −4.50%, 95% CI −6.60 to −2.40%; *p* < 0.001), and in serum liver enzyme levels. The meta‐analysis also found that GLP‐1RA use was significantly associated with weight loss (~4.5 kg) and a decrease in haemoglobin A1c levels (−1.3% or −8 mmol/mol) compared to placebo. Our meta‐regression analyses also showed that sex, age, BMI and T2DM status at baseline, as well as changes in body weight during the trial (although this last factor was found to be marginally significant), did not influence the observed GLP‐1RA‐induced improvement in liver fat content. However, these latter results should be interpreted with caution, as fewer than 10 RCTs were included in meta‐regression analyses.

The evaluation of the RCTs included in the meta‐analysis reveals a paucity of large, high‐quality RCTs with a sufficiently long duration and liver biopsy data, which is the reference method for assessing drug‐induced resolution of MASH or ≥ 1‐stage liver fibrosis improvement. To date, only four placebo‐controlled RCTs (three Phase 2 trials and one Phase 3 trial) have examined the hepatic efficacy of GLP‐1RAs (subcutaneous liraglutide or semaglutide) on MASH resolution and fibrosis improvement. These are the two liver histological endpoints requested by the FDA for the conditional approval of a drug candidate for the treatment of MASH, and they are the histological features most closely associated with the risk of dying or developing liver‐related and extrahepatic complications in MASLD [4, 31].

That said, compared to other previously published meta‐analyses of RCTs examining the effect of GLP‐1RAs in MASLD/MASH (including one from our group) [13, 14, 32, 33, 34], it should be noted that the inclusion of the recent Phase 3 ESSENCE trial, which compares once‐weekly subcutaneous semaglutide 2.4 mg versus placebo for 72 weeks in participants with MASH and fibrosis stages 2 or 3 [15], has allowed us to show, for the first time, a significant improvement in at least one stage of liver fibrosis without worsening of MASH. This finding has not been previously demonstrated in any earlier meta‐analysis of Phase 2 MASH clinical trials using GLP‐1RAs alone, or in other recently published meta‐analyses that included not only GLP‐1RAs but also dual or triple incretin receptor agonists, such as tirzepatide, survodutide, efinopegdutide, or retatrutide [35]. The benefit of GLP‐1RAs (primarily semaglutide 2.4 mg/week) on the severity of liver fibrosis is clinically relevant and is also supported by significant improvements in non‐invasive fibrosis tests (such as ELF score, serum PRO‐C3 levels, and liver stiffness by vibration‐controlled transient elastography). Long‐term data from part 2 of the ESSENCE trial are ongoing to evaluate the effect of semaglutide 2.4 mg weekly on the risk of liver‐related clinical events over 240 weeks [36].

Compelling evidence shows that GLP‐1RAs offer clinical benefits that extend beyond long‐term glycemic control. These antihyperglycemic agents provide significant advantages in reducing the risk of all‐cause mortality and adverse cardiovascular and renal complications for patients with T2DM and obesity [9, 10, 11, 12]. These findings further support the long‐term use of GLP‐1RAs in individuals living with T2DM and MASLD.

The findings of this meta‐analysis may have significant clinical implications. From a clinical perspective, we believe that the evidence supporting the hepatic effectiveness of GLP‐1RAs in resolving MASH, along with the improvement in liver fibrosis, is clinically important, especially considering the growing burden of MASLD and MASH worldwide and the adverse effects of these conditions on the risk of long‐term liver‐related and extrahepatic cardiometabolic and malignant outcomes [3, 4, 5]. In these RCTs, GLP‐1RAs were generally well‐tolerated, with adverse event rates not exceeding those of the placebo, except for a higher frequency of transient, mild‐to‐moderate gastrointestinal disorders. Notably, real‐world retrospective observational cohort studies (involving about 1.5 million patients with T2DM) using an emulated target trial design have also shown that compared to DPP‐4 inhibitors or other antihyperglycaemic agents, the use of GLP‐1RAs is associated with a significantly lower risk of liver‐related clinical events, such as incident cirrhosis, hepatic decompensation events, or hepatocellular carcinoma [37]. GLP‐1RAs align with the American Diabetes Association recommendations as the preferred option for adults with T2DM and MASH, or those at high risk of liver fibrosis [38, 39]. Other Phase 2 and Phase 3 MASH therapeutic trials are also ongoing, investigating the effects of tirzepatide (a dual GLP‐1/GIP receptor co‐agonist) and other dual or triple incretin receptor agonists on liver histological endpoints and long‐term liver‐related clinical events [40, 41, 42].

A detailed discussion of the potential mechanisms by which GLP‐1RAs could exert their beneficial effects on MASLD/MASH is beyond the scope of this meta‐analysis. Nonetheless, these mechanisms are complex and not fully understood. It is reasonable to assume that the hepatoprotective effects of GLP‐1RAs are multifactorial, resulting from their combined positive effects on hyperglycaemia, insulin resistance, and overweight/obesity, as well as their positive effects on the liver, which can be at least partly independent of weight loss [42, 43].

The main strength of this study is its use of a systematic review method to identify all relevant RCTs (published up to April 30, 2025) that meet the predefined inclusion criteria. This provides the most current assessment of the hepatic effectiveness of GLP‐1RAs, including the newest MASH therapeutic trials (the ESSENCE trial). Additionally, most of the included RCTs used MRI‐PDFF or MRS, which are currently the two most accurate imaging techniques for measuring changes in liver fat content, although their accuracy in assessing MASH and liver fibrosis stage is somewhat limited [44]. Finally, the results of the four placebo‐controlled RCTs with liver biopsy data showed no heterogeneity among the studies included in the meta‐analysis (*I* ^2^ = 0%), supporting the notion that the hepatic effects of GLP‐1RAs on MASH resolution and fibrosis improvement are consistently effective in a clinical context.

Our meta‐analysis has important limitations inherent in the included RCTs. First, as previously mentioned, most eligible RCTs had relatively small sample sizes and relatively short treatment durations. Second, only one Phase 3 placebo‐controlled RCT (the ESSENCE trial) with liver histological endpoints as the primary outcome was available for the meta‐analysis. Third, most RCTs enrolled overweight or obese individuals with MASLD and T2DM (predominantly of White race), highlighting the urgent need for RCTs involving people without T2DM and non‐obese individuals with MASLD from diverse ethnic backgrounds. Finally, because of significant sex‐related differences in the prevalence, risk factors, and clinical outcomes of MASLD/MASH [45], future adequately powered RCTs should be designed to evaluate sex‐related differences in the response rates of MASLD/MASH to GLP‐1RA treatment.

In conclusion, the results of this updated meta‐analysis strongly support the hepatic effectiveness of GLP‐1RAs (mainly semaglutide 2.4 mg/week) in reducing liver fat content, achieving resolution of MASH, and improving liver fibrosis in individuals with MASH and fibrosis, regardless of T2DM status. While we await results from Phase 3 randomised controlled trials designed to evaluate the long‐term benefits of GLP‐1RAs on liver‐related clinical events, these findings suggest that GLP‐1RAs are a suitable treatment option (either alone or combined with other liver‐directed pharmacotherapies, such as resmetirom, peroxisome proliferator‐activated‐receptor agonists, or FGF‐21 analogues) for individuals living with MASLD or MASH, especially among those who are obese or have T2DM.

## Author Contributions

A.M. and G.T. were involved in the conception of the study, as well as the analysis and interpretation of the results. G.T. wrote the first draft of the manuscript. A.M., R.M., V.F., M.G.L. and G.T. were involved in the conduct of the study and searched the published articles. N.S., H.T. and C.D.B. were involved in the interpretation of the results and contributed to the discussion. All authors edited, reviewed, and approved the final version of the manuscript. G.T. is the guarantor of this work and, as such, has full access to all the data in the study and takes responsibility for the integrity of the data and the accuracy of the data analysis.

## Ethics Statement

This study involves human participants but was not approved by an Ethics Committee(s) or Institutional Board(s). In fact, approval from an Ethics Committee is unnecessary because this is a meta‐analysis of published observational studies that have already obtained informed consent from participants and received ethical approval from their local Ethics committees.

## Conflicts of Interest

The authors did not report any disclosures related to this work. C.D.B. has received research grants from Echosens. C.D.B. is supported in part by the Southampton National Institute for Health Research (NIHR) Biomedical Research Centre (NIHR203319). N.S. received fees for consultancy and giving scientific talks from Allergan, AstraZeneca, Boehringer Ingelheim, Gilead, Genkyotex, GSK, Intercept Pharma, Lilly, Merck Sharpe & Dohme, Novartis, Novo Nordisk, Pfizer, and Sanofi; and received research support from AstraZeneca, Sanofi, DSM. Nutritional Products, and Roche Diagnostics. G.T. is supported in part by grants from the University School of Medicine of Verona, Verona, Italy.

## Supporting information


**Data S1:** liv70256‐sup‐0001‐supinfo.pdf.

## Data Availability

The data that supports the findings of this study are available in the Supporting Information of this article.
