# Supplementary material for: Glucagon‐Like Peptide‐1 Receptor Agonists Improve MASH and Liver Fibrosis: A Meta‐Analysis of Randomised Controlled Trials
Source: Liver Int. 2025 Jul 30;45(9):e70256. doi: 10.1111/liv.70256 (PMC12309150; doi:10.1111/liv.70256)

ONLINE-ONLY SUPPLEMENTARY MATERIAL

Supplementary Table S1. Studies excluded *at the eligibility stage* according to the PRISMA flow diagram.

Authors	Year	Main reason(s)
Alkhouri al.	2022	Randomised, open-label phase 2 trial evaluating the safety and efficacy of semaglutide in combination with cilofexor and firsocostat for 24 weeks in patients with MASH
Romero-Gómez et al.	2023	Phase 2a active-comparator-controlled trial to evaluate the efficacy and safety of efinopegdutide vs. semaglutide for 24 weeks in patients with MASLD
Harrison et al.	2025	Randomised, phase 2b trial evaluating the safety and efficacy of efruxifermin in combination with a GLP-1 receptor agonist for 12 weeks in patients with T2D and MASH

References

1.Alkhouri N, Herring R, Kabler H, Kayali Z, Hassanein T, Kohli A, Huss RS, Zhu Y, Billin AN, Damgaard LH, Buchholtz K, Kjær MS, Balendran C, Myers RP, Loomba R, Noureddin M. Safety and efficacy of combination therapy with semaglutide, cilofexor and firsocostat in patients with non-alcoholic steatohepatitis: A randomised, open-label phase II trial. J Hepatol. 2022;77:607-618.
2.Romero-Gómez M, Lawitz E, Shankar RR, Chaudhri E, Liu J, Lam RLH, Kaufman KD, Engel SS; MK-6024 P001 Study Group. A phase IIa active-comparator-controlled study to evaluate the efficacy and safety of efinopegdutide in patients with non-alcoholic fatty liver disease. J Hepatol. 2023;79:888-897.
3.Harrison SA, Frias JP, Lucas KJ, Reiss G, Neff G, Bollepalli S, Su Y, Chan D, Tillman EJ, Moulton A, de Temple B, Zari A, Shringarpure R, Rolph T, Cheng A, Yale K. Safety and Efficacy of Efruxifermin in Combination With a GLP-1 Receptor Agonist in Patients With NASH/MASH and Type 2 Diabetes in a Randomized Phase 2 Study. Clin Gastroenterol Hepatol. 2025;23:103-113.

Supplementary Table S2. Evaluation of bias risk for each eligible RCT assessed using the Cochrane Collaboration's tool.

Authors	Year	Random Sequence Generation (Selection Bias)	Allocation Concealment (Selection Bias)	Blinding of Participants and Personnel (Performance Bias)	Blinding of Outcome Assessment (Detection Bias)	Incomplete Outcome Data (Attrition Bias)	Selective Reporting (Reporting Bias)	Other Bias
**Armstrong et al.**	**2016**	Low	Low	Low	Low	Low	Low	Low
**Dutour et al.**	**2016**	Low	Low	Low	Low	Unclear	Unclear	Unclear
**Frossing et al.**	**2018**	Low	Low	Low	Low	Unclear	Unclear	Unclear
**Yan et al.**	**2019**	Low	Low	Unclear	Low	Unclear	Unclear	Unclear
**Khoo et al.**	**2019**	Low	Low	Unclear	Low	Low	Unclear	Unclear
**Liu et al.**	**2020**	Low	Low	Unclear	Low	Unclear	Unclear	Unclear
**Bizino et al.**	**2020**	Low	Low	Low	Low	Low	Low	Unclear
**Kuchay et al.**	**2020**	Low	Low	Unclear	Low	Low	Low	Unclear
**Newsome et al.**	**2020**	Low	Low	Low	Low	Low	Low	Low
**Guo et al.**	**2020**	Low	Low	Unclear	Low	Low	Low	Unclear
**Flint et al.**	**2021**	Low	Low	Low	Low	Low	Low	Low
**Loomba et al.**	**2023**	Low	Low	Low	Low	Low	Low	Low
**Sanyal et al.**	**2025**	Low	Low	Low	Low	Low	Low	Low

Note: For each of the seven domains of the Cochrane Collaboration's tool, the presence of low risk of bias was highlighted in green; unclear risk was highlighted in yellow (no studies had high risk of bias).

Supplementary Figure S1. The PRISMA flow diagram illustrates the search and selection processes for the meta-analysis.

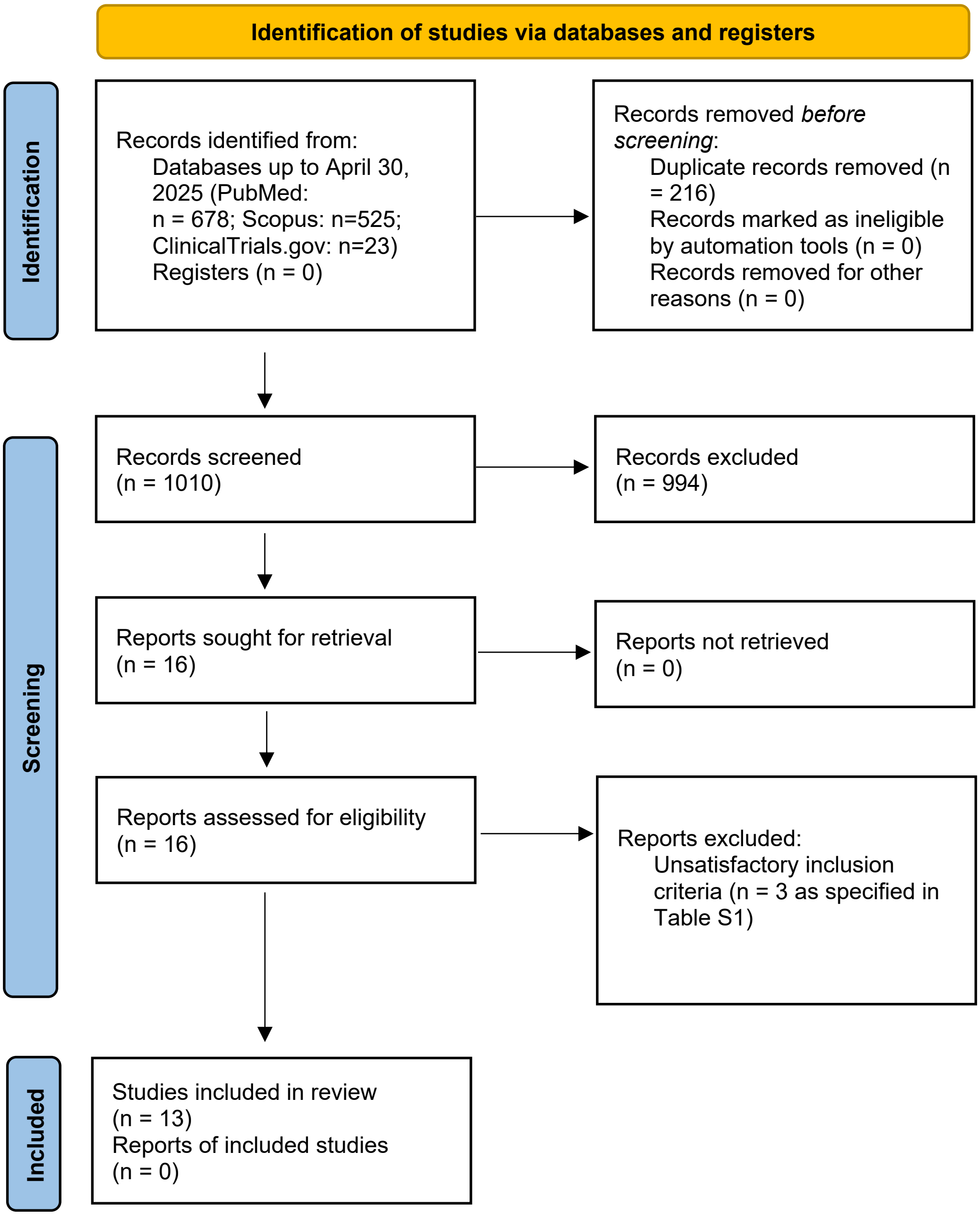

Supplementary Figure S2. Forest plot and pooled estimates of the effect of GLP-1RAs on serum alanine aminotransferase levels compared to placebo or reference therapy.

Study or subgroup	Mean (SD) / total Experimental	Control	Weight (%), common	Weight (%), random	Mean Difference, IV, common + random (95% CI)
Armstrong, 2016, Liraglutide (1.8 mg)	-26.60 (34.4000) / 23	-10.20 (35.8000) / 22	1.5	5.4	-16.40 [-36.93; 4.13]
Dutour, 2016, Exenatide (10 ug)	-3.00 (33.0000) / 22	-1.00 (27.0000) / 22	2.0	6.1	-2.00 [-19.82; 15.82]
Frossing, 2017, Liraglutide (1.8 mg)	-1.10 (14.3000) / 48	-3.10 (14.3000) / 24	12.9	9.5	2.00 [-5.01; 9.01]
Yan, 2019, Liraglutide (1.8 mg)	-5.20 (12.5000) / 24	-0.80 (18.2000) / 24	8.1	9.0	-4.40 [-13.23; 4.43]
Khoo, 2019, Liraglutide (1.8 mg)	-26.00 (33.0000) / 15	-39.00 (35.0000) / 15	1.1	4.5	13.00 [-11.34; 37.34]
Liu, 2020, Exenatide (10 ug)	-22.10 (21.4000) / 38	-9.10 (20.8000) / 38	7.0	8.8	-13.00 [-22.49; -3.51]
Kuchay, 2020, Dulaglutide (1.5 mg)	-26.40 (28.6000) / 27	-13.90 (25.0000) / 25	3.0	7.1	-12.50 [-27.07; 2.07]
Bizino, 2020, Liraglutide (1.8 mg)	16.00 (12.0000) / 23	14.00 (10.0000) / 26	16.3	9.7	2.00 [-4.23; 8.23]
Newsome, 2020, Semaglutide (0.1 mg)	-21.00 (90.0000) / 80	-11.00 (92.0000) / 80	0.8	3.8	-10.00 [-38.20; 18.20]
Newsome, 2020, Semaglutide (0.2 mg)	-21.30 (78.0000) / 78	-11.00 (92.0000) / 80	0.9	4.1	-10.30 [-36.87; 16.27]
Newsome, 2020, Semaglutide (0.4 mg)	-31.00 (84.0000) / 82	-11.00 (92.0000) / 80	0.9	4.0	-20.00 [-47.15; 7.15]
Guo, 2020, Liraglutide (1.8 mg)	-6.00 (14.5000) / 31	0.00 (0.7000) / 30	24.2	10.0	-6.00 [-11.11; -0.89]
Loomba, 2023, Semaglutide (2.4 mg)	-16.00 (21.2000) / 47	18.10 (23.8000) / 24	5.0	8.2	-34.10 [-45.39; -22.81]
Sanyal, 2025, Semaglutide (2.4 mg)	-38.00 (41.0000) / 534	-12.00 (42.7000) / 266	16.5	9.7	-26.00 [-32.20; -19.80]
Total (95% CI), common	1072	756	100.0	.	-8.97 [-11.49; -6.46]
Total (95% CI), random			.	100.0	-9.99 [-16.80; -3.18]

Heterogeneity: τ^2=114.0128; χ^2=77.47, df=13, P < 0.0001; I^2=83.2%

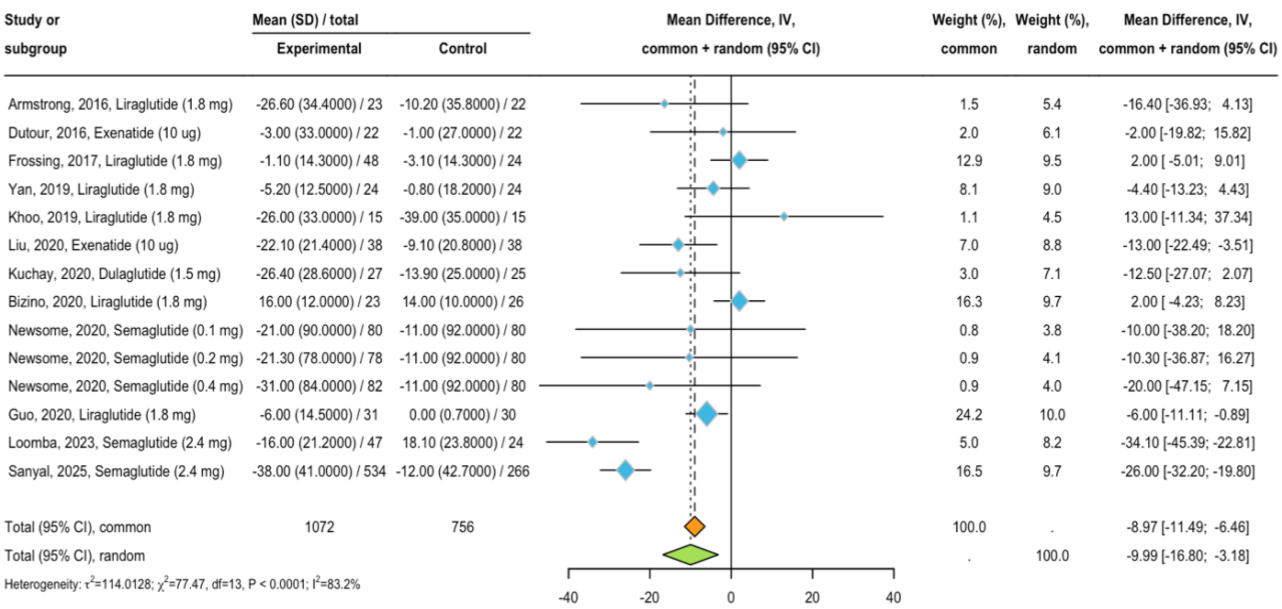

Supplementary Figure S3. Forest plot and pooled estimates of the effect of GLP-1RAs on serum aspartate aminotransferase levels compared to placebo or reference therapy.

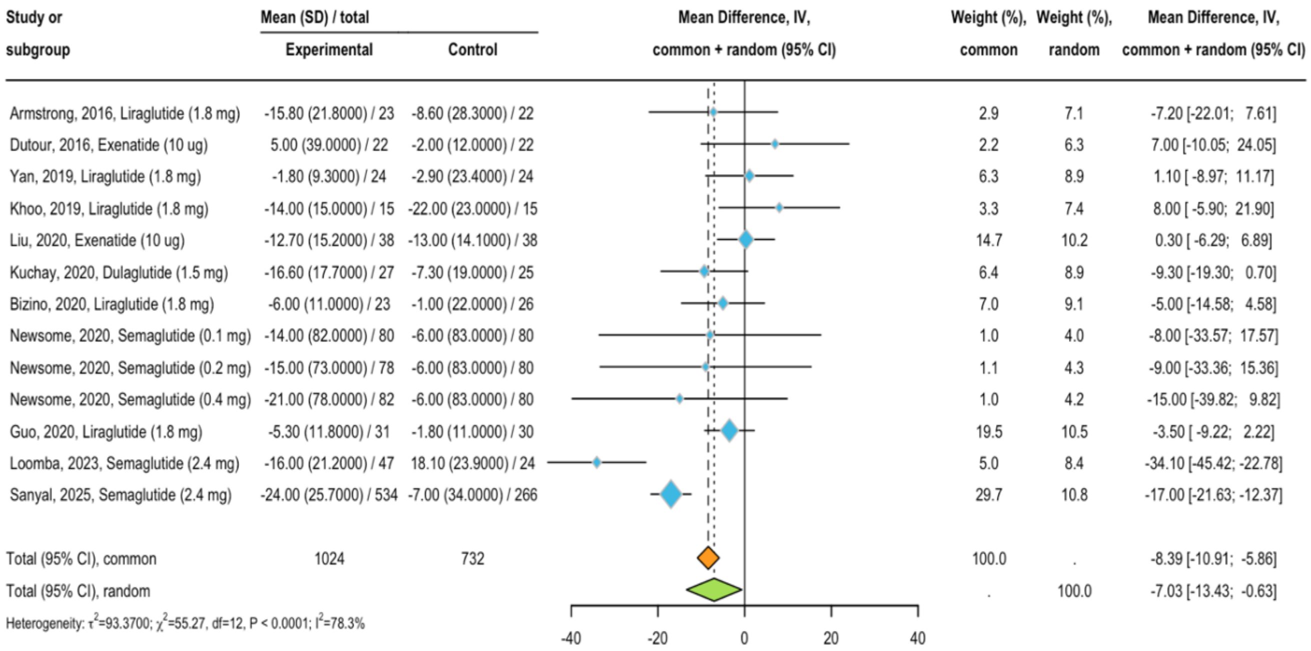

Supplementary Figure S4. Forest plot and pooled estimates of the effect of GLP-1RAs on serum gamma-glutamyltransferase levels compared to placebo or reference therapy.

Study or subgroup	Mean (SD) / total Experimental	Control	Weight (%), common	Weight (%), random	Mean Difference, IV, common + random (95% CI)
Armstrong, 2016, Liraglutide (1.8 mg)	-33.70 (42.5000) / 23	-7.20 (28.3000) / 22	5.8	10.7	-26.50 [-47.51; -5.49]
Dutour, 2016, Exenatide (10 ug)	-13.00 (41.0000) / 22	-3.00 (27.0000) / 22	6.1	10.9	-10.00 [-30.51; 10.51]
Liu, 2020, Exenatide (10 ug)	-23.20 (13.7000) / 38	-5.00 (70.2000) / 38	5.0	10.0	-18.20 [-40.94; 4.54]
Kuchay, 2020, Dulaglutide (1.5 mg)	-19.40 (17.7000) / 27	-6.30 (23.0000) / 25	20.5	14.8	-13.10 [-24.32; -1.88]
Bizino, 2020, Liraglutide (1.8 mg)	1.00 (18.0000) / 23	-3.00 (11.0000) / 26	35.9	15.9	4.00 [-4.48; 12.48]
Newsome, 2020, Semaglutide (0.1 mg)	-15.00 (96.0000) / 80	-2.00 (96.0000) / 80	2.9	7.7	-13.00 [-42.75; 16.75]
Newsome, 2020, Semaglutide (0.2 mg)	-22.00 (103.0000) / 78	-2.00 (96.0000) / 80	2.7	7.3	-20.00 [-51.06; 11.06]
Newsome, 2020, Semaglutide (0.4 mg)	-33.00 (105.0000) / 82	-2.00 (96.0000) / 80	2.7	7.3	-31.00 [-61.97; -0.03]
Loomba, 2023, Semaglutide (2.4 mg)	-29.90 (103.5000) / 47	12.20 (250.3000) / 24	0.2	1.0	-42.10 [-146.52; 62.32]
Sanyal, 2025, Semaglutide (2.4 mg)	-49.00 (78.1000) / 534	-7.00 (82.5000) / 266	18.2	14.5	-42.00 [-53.92; -30.08]
Total (95% CI), common	954	663	100.0	.	-13.80 [-18.88; -8.72]
Total (95% CI), random			.	100.0	-18.29 [-29.26; -7.32]

Heterogeneity: τ^2=178.6862; χ^2=41.71, df=9, P < 0.0001; I^2=78.4%

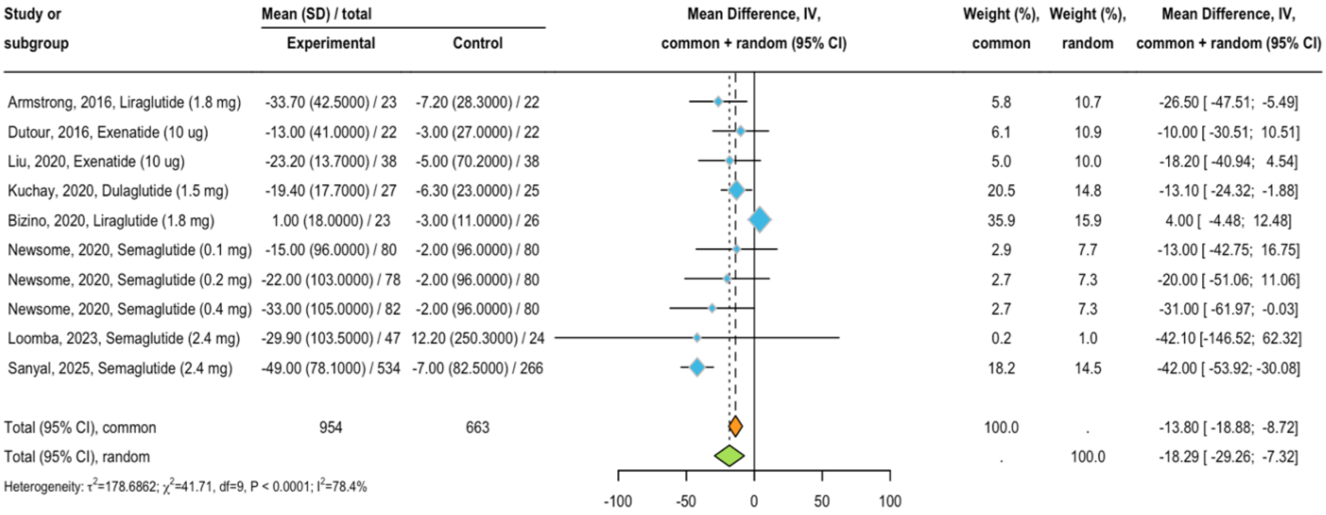

Supplementary Figure S5. Forest plot and pooled estimates of the effect of GLP-1RAs on body weight compared to placebo or reference therapy.

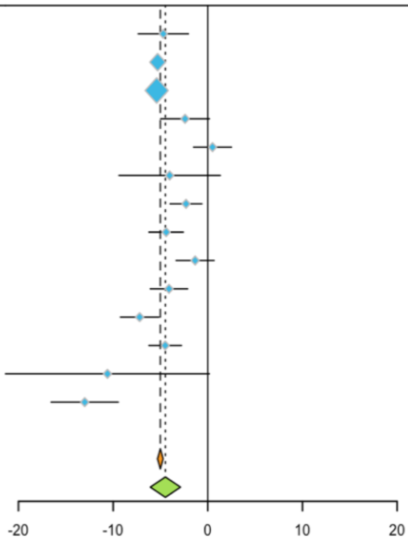

Study or subgroup	Mean (SD) / total: Experimental	Mean (SD) / total: Control	Weight (%), common	Weight (%), random	Mean Difference, IV, common + random (95% CI)
Armstrong, 2016, Liraglutide (1.8 mg)	-5.30 (4.7000) / 23	-0.60 (4.4000) / 22	1.3	7.2	-4.70 [-7.36; -2.04]
Dutour, 2016, Exenatide (10 ug)	-5.50 (1.2000) / 22	-0.20 (0.8000) / 22	24.9	8.8	-5.30 [-5.90; -4.70]
Frossing, 2017, Liraglutide (1.8 mg)	-5.20 (0.7000) / 48	0.20 (0.9000) / 24	53.5	8.9	-5.40 [-5.81; -4.99]
Yan, 2019, Liraglutide (1.8 mg)	-3.60 (4.9000) / 24	-1.20 (4.2000) / 24	1.4	7.2	-2.40 [-4.98; 0.18]
Khoo, 2019, Liraglutide (1.8 mg)	-3.00 (2.2000) / 15	-3.50 (3.3000) / 15	2.2	7.8	0.50 [-1.51; 2.51]
Liu, 2020, Exenatide (10 ug)	-5.35 (9.7900) / 38	-1.32 (13.7000) / 38	0.3	4.5	-4.03 [-9.38; 1.32]
Kuchay, 2020, Dulaglutide (1.5 mg)	-4.30 (2.6000) / 27	-2.00 (3.5000) / 25	3.2	8.1	-2.30 [-3.99; -0.61]
Bizino, 2020, Liraglutide (1.8 mg)	-4.30 (3.8000) / 23	0.10 (2.5000) / 26	2.7	8.0	-4.40 [-6.23; -2.57]
Newsome, 2020, Semaglutide (0.1 mg)	-3.24 (6.4000) / 80	-1.90 (6.6000) / 80	2.2	7.8	-1.34 [-3.35; 0.67]
Newsome, 2020, Semaglutide (0.2 mg)	-6.00 (6.1000) / 78	-1.90 (6.6000) / 80	2.3	7.8	-4.10 [-6.08; -2.12]
Newsome, 2020, Semaglutide (0.4 mg)	-9.10 (6.6000) / 82	-1.90 (6.6000) / 80	2.2	7.8	-7.20 [-9.23; -5.17]
Guo, 2020, Liraglutide (1.8 mg)	-5.10 (4.3000) / 31	-0.60 (2.3000) / 30	3.0	8.1	-4.50 [-6.22; -2.78]
Loomba, 2023, Semaglutide (2.4 mg)	-10.20 (19.1100) / 47	0.40 (23.2000) / 24	0.1	1.8	-10.60 [-21.37; 0.17]
Sanyal, 2025, Semaglutide (2.4 mg)	-15.00 (23.5000) / 534	-2.00 (24.5000) / 266	0.7	6.2	-13.00 [-16.56; -9.44]
Total (95% CI), common	1072	756	100.0	.	-5.01 [-5.31; -4.71]
Total (95% CI), random			.	100.0	-4.48 [-6.08; -2.88]

Heterogeneity: τ^2=7.4535; χ^2=86.54, df=13, P < 0.0001; I^2=85.0%

Supplementary Figure S6. Forest plot and pooled estimates of the effect of GLP-1RAs on hemoglobin A1c levels compared to placebo or reference therapy.

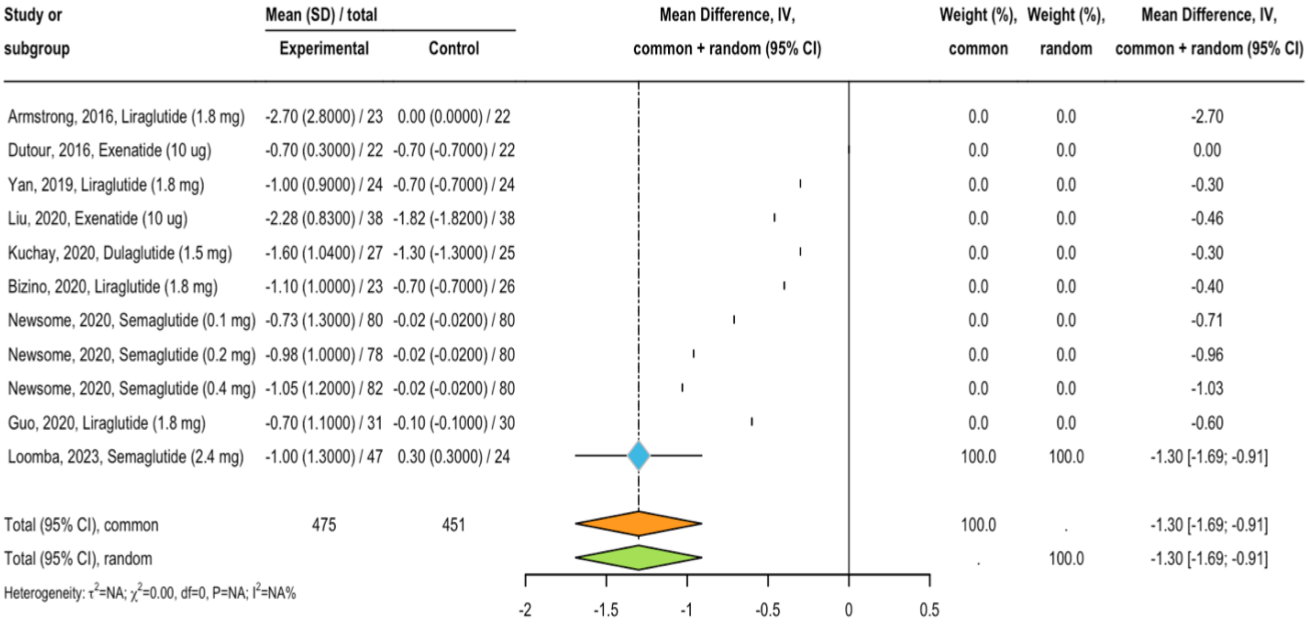

Supplementary Figure S7. Forest plot and pooled estimates of the effect of GLP1RAs on MRI-assessed liver fat content compared to placebo or reference therapy in eligible RCTs, stratified by study country.

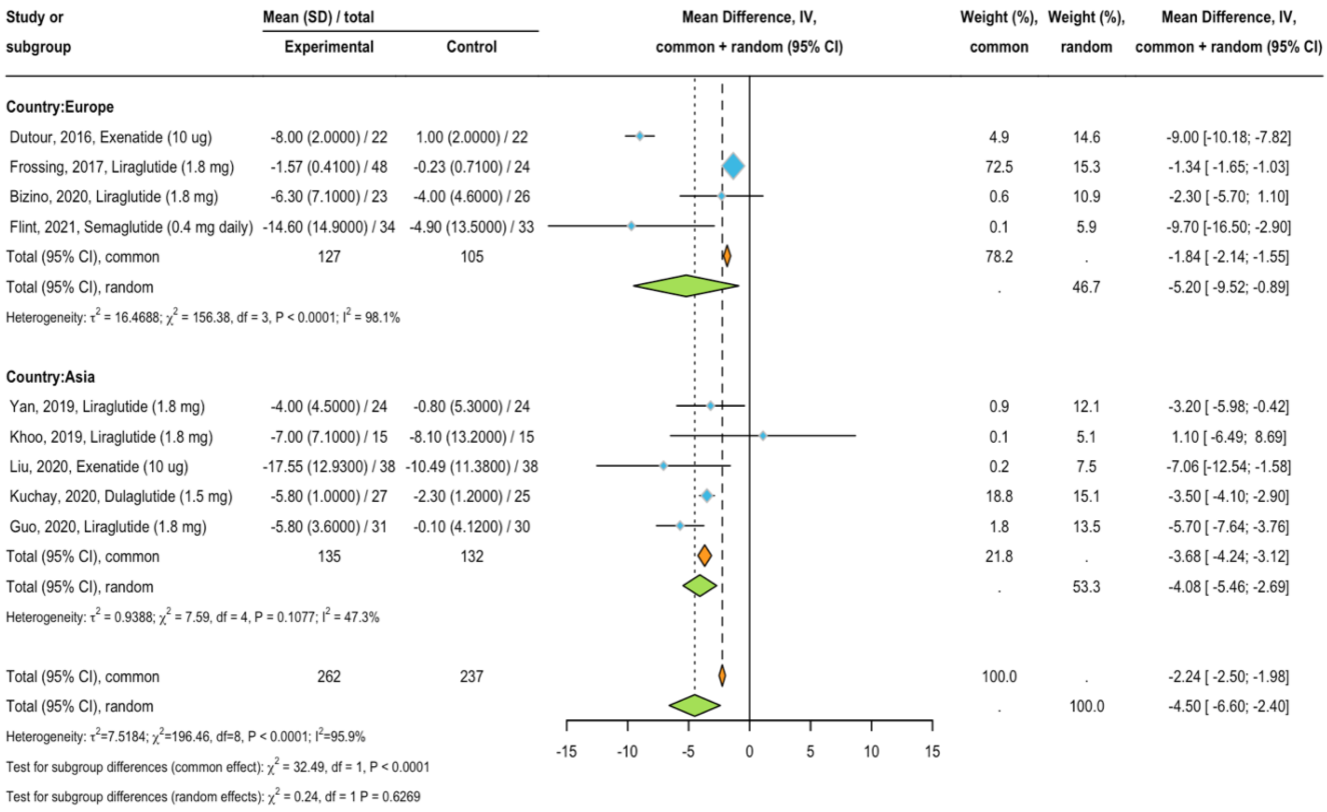

Supplementary Figure S8. Univariable meta-regression analysis. This bubble plot features a fixed meta-regression line (in blue) representing the pooled estimates of the effect of GLP-1RAs on the mean difference (MD) of liver fat content by age in RCTs employing magnetic resonance-based techniques.

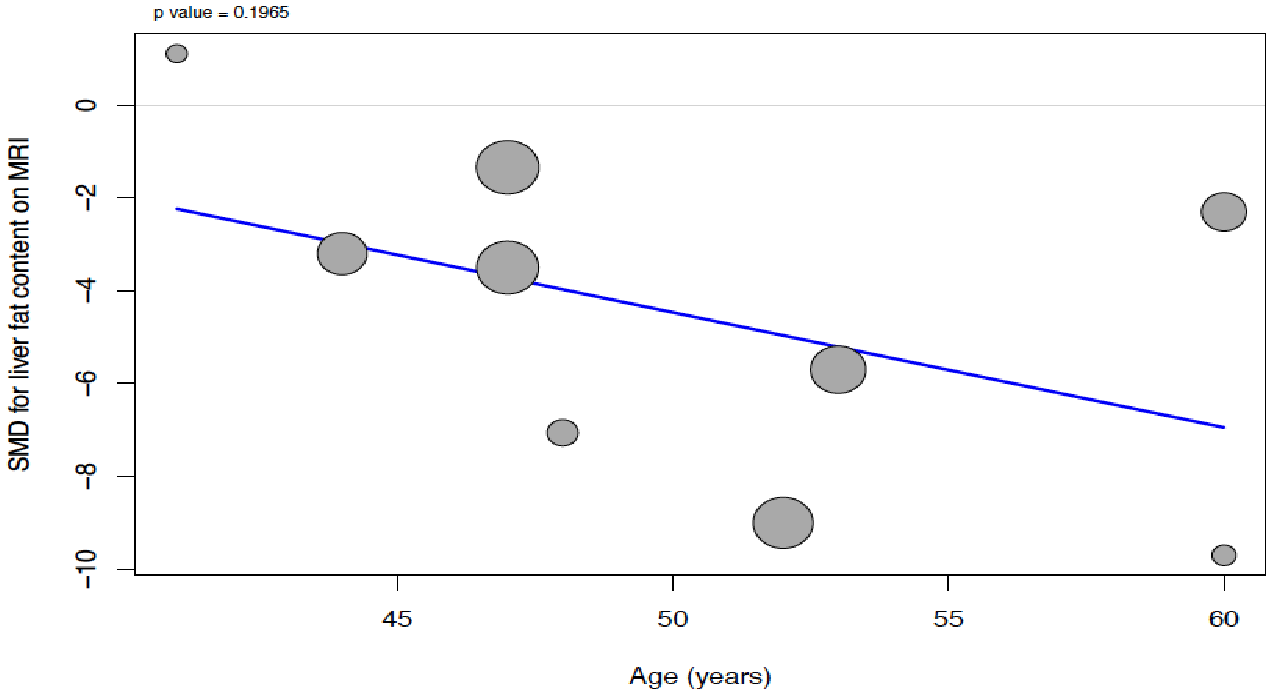

Supplementary Figure S9: Univariable meta-regression analysis. Bubble plot with a fixed meta-regression line (in blue) illustrating the pooled estimates of the effect of GLP-1RAs on the mean difference (MD) in liver fat content by male sex in RCTs utilizing magnetic resonance-based techniques.

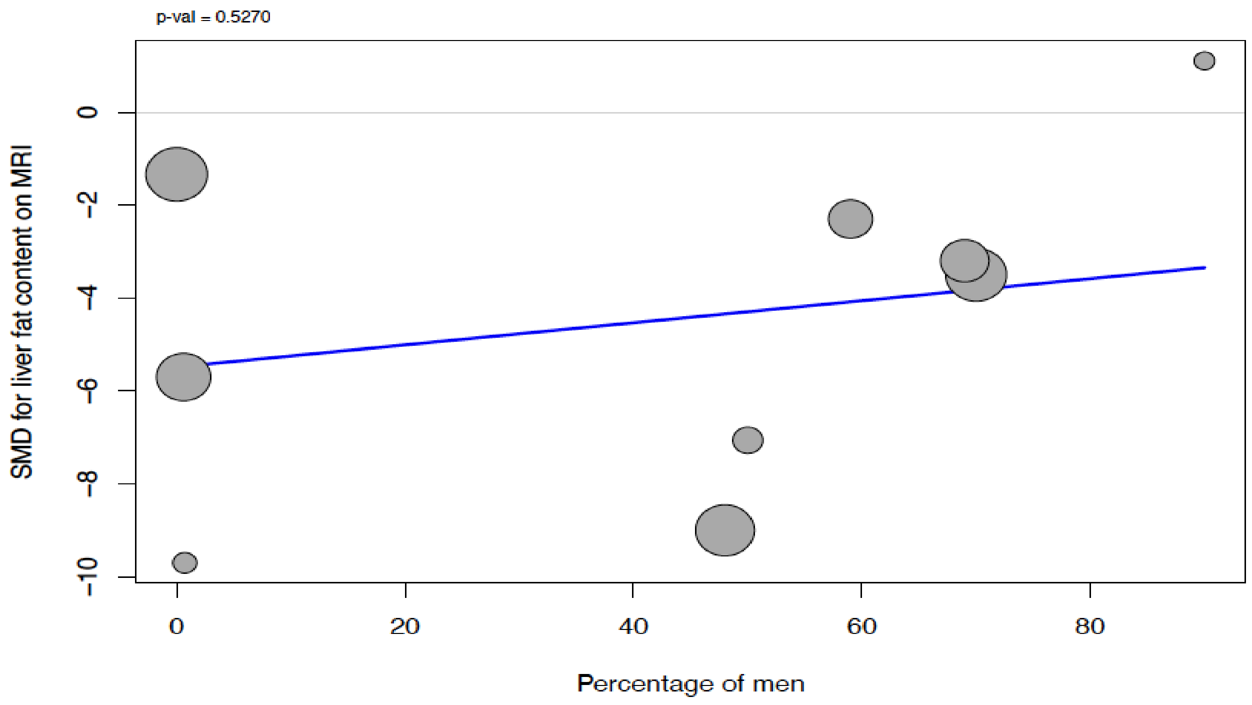

Supplementary Figure S10. Univariable meta-regression analysis. The bubble plot displays a fixed meta-regression line (in blue) regarding the pooled estimates of the effect of GLP-1RAs on the mean difference (MD) of liver fat content by body mass index (BMI) in RCTs utilizing magnetic resonance-based techniques.

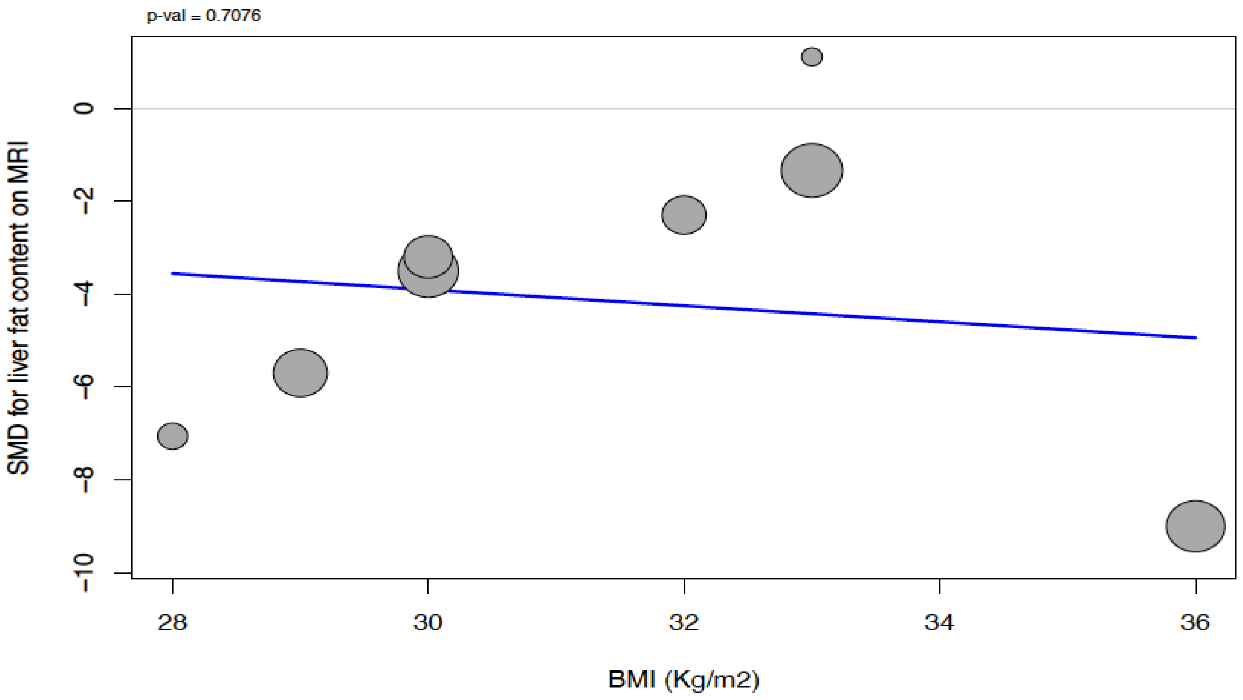

Supplementary Figure S11. Univariable meta-regression analysis. Bubble plot with a fixed meta-regression line (in blue) regarding the pooled estimates of the effect of GLP-1RAs on the mean difference (MD) in liver fat content by percentage of type 2 diabetes at baseline in RCTs utilizing magnetic resonance-based techniques.

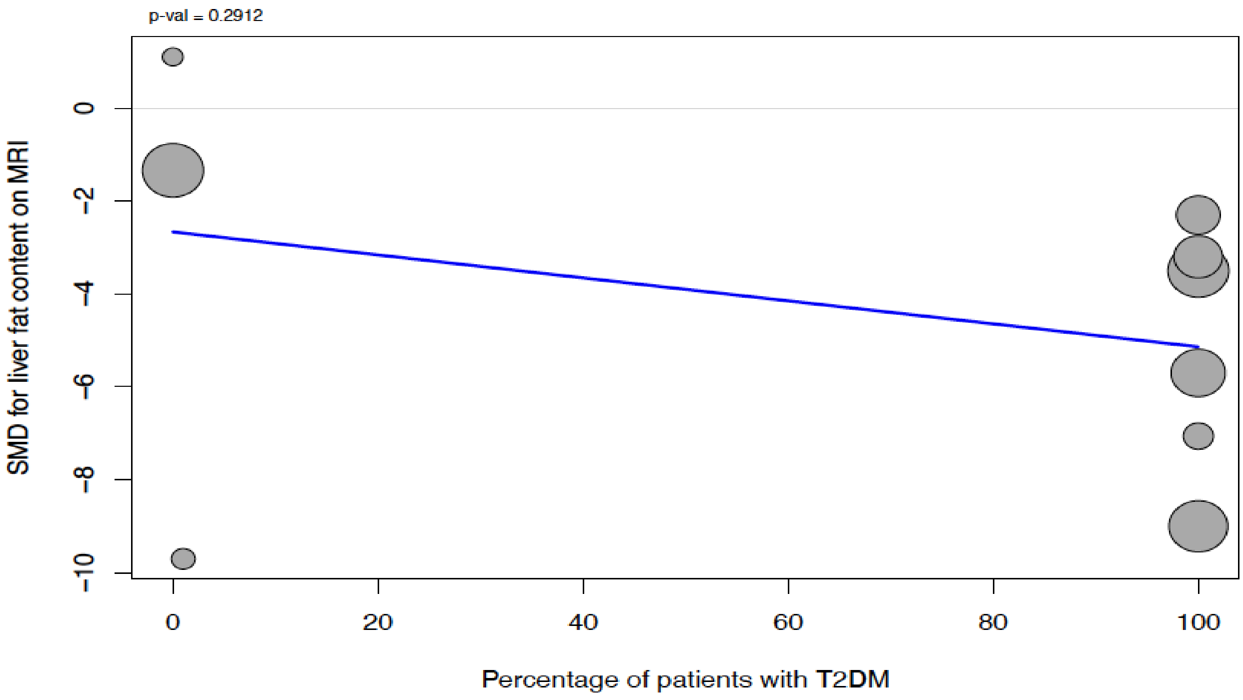

Supplementary Figure S12. Univariable meta-regression analysis. Bubble plot with a fixed meta-regression line (in blue) regarding the pooled estimates of the effect of GLP-1RAs on the mean difference (MD) in liver fat content by percentage changes in body weight during the trial in RCTs utilizing magnetic resonance-based techniques.

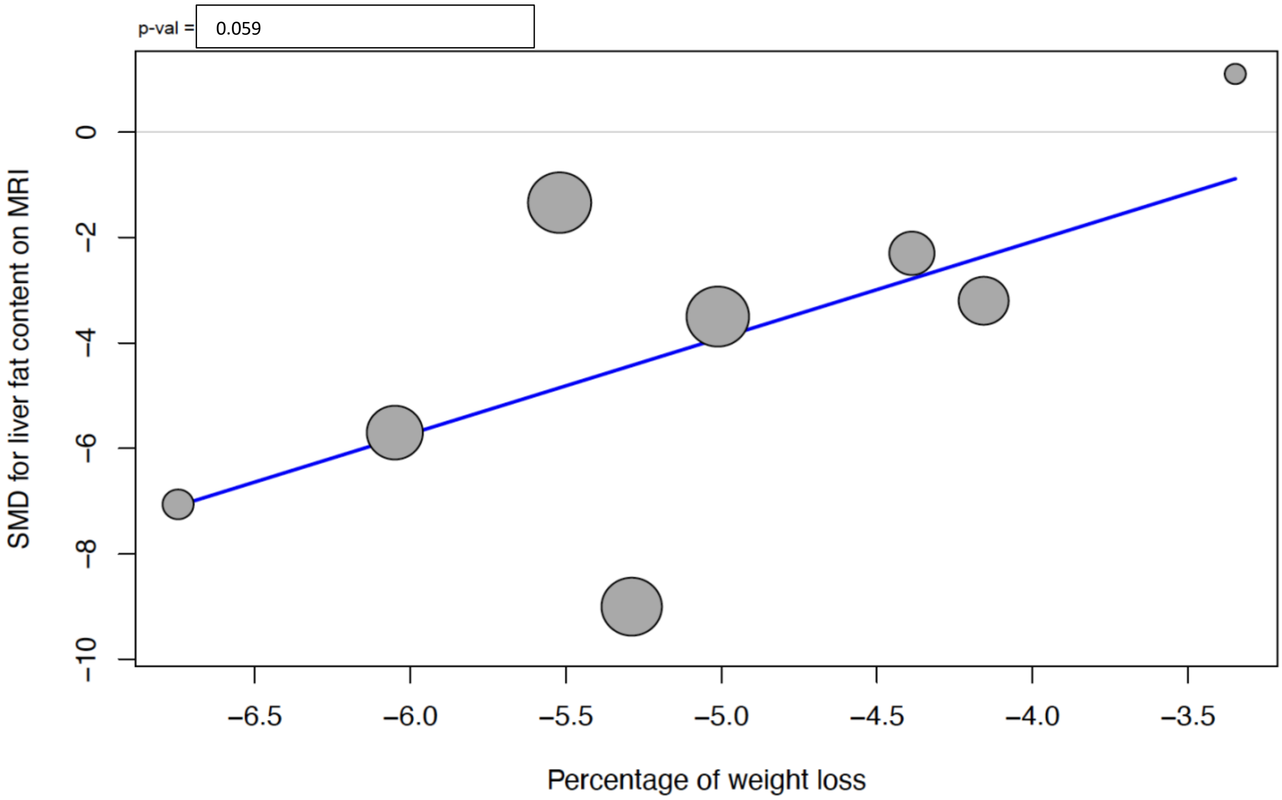

Supplementary Figure S13. Influence analysis in meta-analysis using leave-one-out method.

Panel A) Influence analysis in meta-analysis using leave-one-out method about the effect of GLP-1RAs on the histologic resolution of MASH without worsening of liver fibrosis, compared to placebo.

	OR	95%-CI	p-value	tau^2	tau	I^2
Omitting Armstrong, 2016, Liraglutide (1.8 mg)	3.4279	[2.6372; 4.4557]	< 0.0001	< 0.0001	0.0023	0%
Omitting Newsome, 2020, Sema (0.1 mg daily)	3.6452	[2.5521; 5.2063]	< 0.0001	0.0257	0.1603	13.1%
Omitting Newsome, 2020, Sema (0.2 mg daily)	3.7786	[2.6197; 5.4502]	< 0.0001	0.0297	0.1722	2.1%
Omitting Newsome, 2020, Sema (0.4 mg daily)	3.2531	[2.4795; 4.2681]	< 0.0001	0	0	0%
Omitting Sanyal, 2025, Sema (2.4 mg weekly)	4.0774	[2.4453; 6.7988]	< 0.0001	0.0298	0.1726	0%
Random effects model	3.4797	[2.6855; 4.5087]	< 0.0001	0	0	0%

Panel B) Influence analysis in meta-analysis using leave-one-out method about the effect of GLP-1RAs on the improvement in ≥1-stage liver fibrosis without worsening of MASH compared to placebo.

	OR	95%-CI	p-value	tau^2	tau	I^2
Omitting Armstrong, 2016, Liraglutide (1.8 mg)	1.7545	[1.3040; 2.3607]	0.0002	0.0103	0.1014	3.1%
Omitting Newsome, 2020, Sema (0.1 mg daily)	1.6803	[1.1577; 2.4388]	0.0063	0.0349	0.1868	3.3%
Omitting Newsome, 2020, Sema (0.2 mg daily)	1.9507	[1.4736; 2.5822]	< 0.0001	0	0	0%
Omitting Newsome, 2020, Sema (0.4 mg daily)	1.7947	[1.2885; 2.4999]	0.0005	0.0169	0.1298	0%
Omitting Sanyal, 2025, Sema (2.4 mg weekly)	1.4956	[0.9791; 2.2847]	0.0626	0	0	0%
Random effects model	1.7949	[1.3731; 2.3465]	< 0.0001	0.0014	0.0376	0%

Panel C) Influence analysis in meta-analysis using leave-one-out method about the effect of GLP-1RAs on the absolute percentage of liver fat content, assessed by magnetic resonance-based techniques.

	MD	95%-CI	p-value	tau^2	tau	I^2
Omitting Dutour, 2016, Exenatide (10 ug)	-3.5340	[-5.1199; -1.9481]	< 0.0001	2.8870	1.6991	89.1%
Omitting Frossing, 2017, Liraglutide (1.8 mg)	-5.0838	[-7.2162; -2.9514]	< 0.0001	6.2905	2.5081	90.7%
Omitting Yan, 2019, Liraglutide (1.8 mg)	-4.6852	[-7.0630; -2.3074]	0.0001	8.6835	2.9468	96.4%
Omitting Liu, 2020, Exenatide (10 ug)	-4.2905	[-6.5351; -2.0459]	0.0002	8.0086	2.8299	96.4%
Omitting Kuchay, 2020, Dulaglutide (1.5 mg)	-4.6797	[-7.1290; -2.2304]	0.0002	9.0036	3.0006	96.0%
Omitting Bizino, 2020, Liraglutide (1.8 mg)	-4.7762	[-7.0835; -2.4689]	< 0.0001	8.1877	2.8614	96.4%
Omitting Khoo, 2019, Liraglutide (1.8 mg)	-4.7992	[-6.9440; -2.6543]	< 0.0001	7.4072	2.7216	96.4%
Omitting Flint, 2021, Semaglutide (0.4 mg daily)	-4.1783	[-6.3199; -2.0367]	0.0001	7.3092	2.7035	96.4%
Omitting Guo, 2020, Liraglutide (1.8 mg)	-4.3216	[-6.7273; -1.9159]	0.0004	8.7758	2.9624	96.2%
Random effects model	-4.5002	[-6.6025; -2.3978]	< 0.0001	7.5184	2.7420	95.9%

Supplementary Figure S14. Funnel plot evaluating the potential for publication bias in RCTs using liver biopsy data.

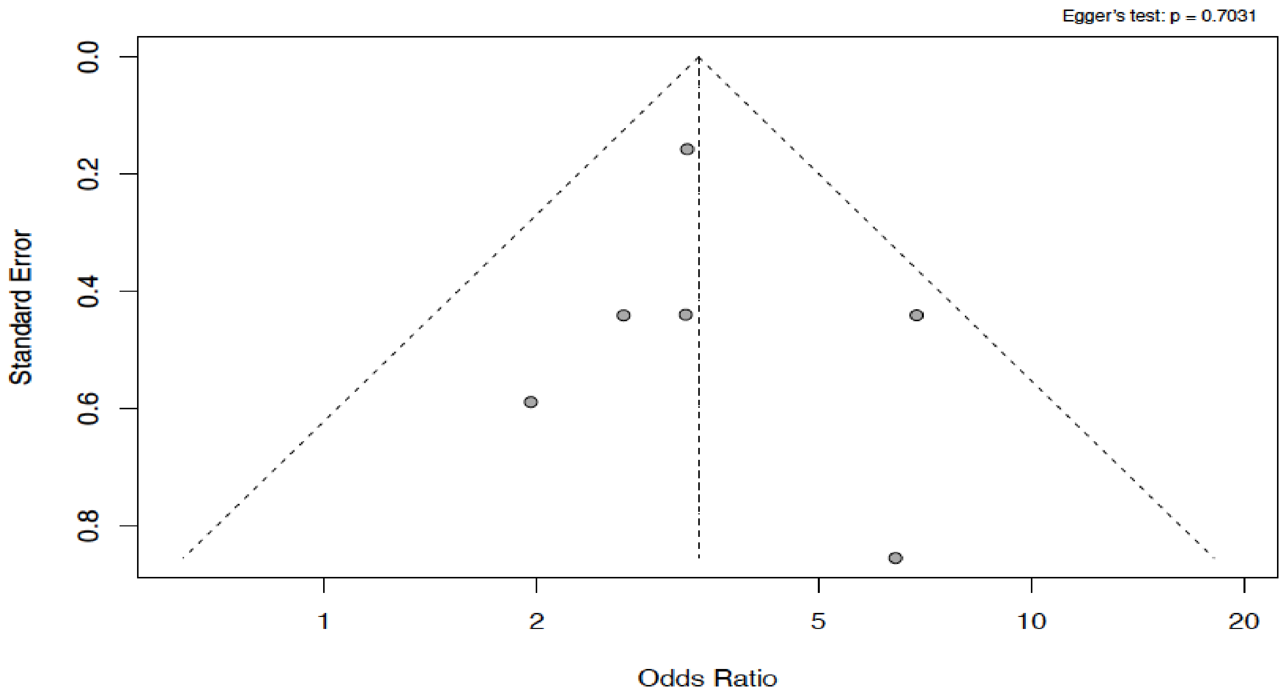

Supplementary Figure S15. Funnel plot assessing the potential for publication bias in RCTs using magnetic resonance-based techniques.

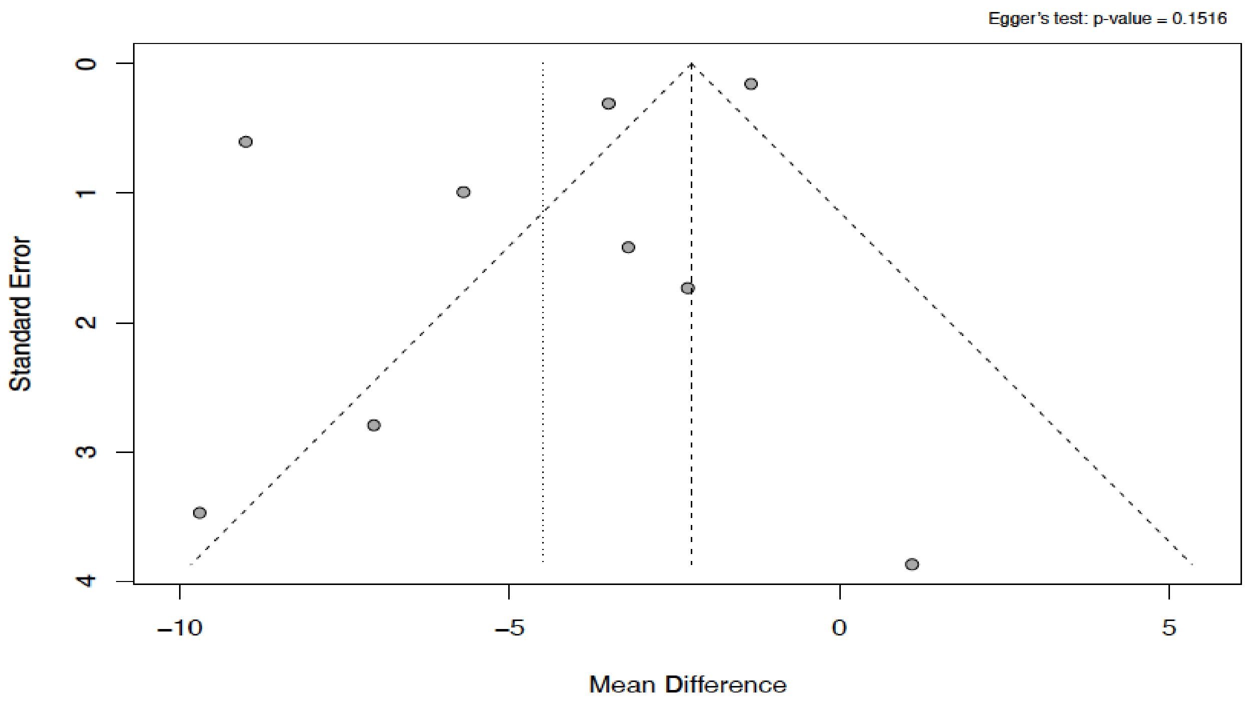

Supplement: Supplementary file 1 — Data S1: liv70256‐sup‐0001‐supinfo.pdf. [file LIV-45-0-s001.pdf]
